# Genetic Features of Reproductive Traits in Bovine and Buffalo: Lessons From Bovine to Buffalo

**DOI:** 10.3389/fgene.2021.617128

**Published:** 2021-03-23

**Authors:** Baoshun Shao, Hui Sun, Muhammad Jamil Ahmad, Nasser Ghanem, Hamdy Abdel-Shafy, Chao Du, Tingxian Deng, Shahid Mansoor, Yang Zhou, Yifen Yang, Shujun Zhang, Liguo Yang, Guohua Hua

**Affiliations:** ^1^Key Lab of Agricultural Animal Genetics, Breeding and Reproduction of Ministry of Education, College of Animal Science and Technology, Huazhong Agricultural University, Wuhan, China; ^2^Department of Animal Production, Faculty of Agriculture, Cairo University, Giza, Egypt; ^3^Guangxi Provincial Key Laboratory of Buffalo Genetics, Breeding and Reproduction Technology, Buffalo Research Institute, Chinese Academy of Agricultural Sciences, Nanning, China; ^4^National Institute for Biotechnology and Genetic Engineering (NIBGE), Faisalabad, Pakistan; ^5^International Joint Research Centre for Animal Genetics, Breeding and Reproduction, Wuhan, China; ^6^Hubei Province’s Engineering Research Center in Buffalo Breeding and Products, Wuhan, China

**Keywords:** reproduction, breeding, genetic improvement, heritability, GWAS

## Abstract

Bovine and buffalo are important livestock species that have contributed to human lives for more than 1000 years. Improving fertility is very important to reduce the cost of production. In the current review, we classified reproductive traits into three categories: ovulation, breeding, and calving related traits. We systematically summarized the heritability estimates, molecular markers, and genomic selection (GS) for reproductive traits of bovine and buffalo. This review aimed to compile the heritability and genome-wide association studies (GWASs) related to reproductive traits in both bovine and buffalos and tried to highlight the possible disciplines which should benefit buffalo breeding. The estimates of heritability of reproductive traits ranged were from 0 to 0.57 and there were wide differences between the populations. For some specific traits, such as age of puberty (AOP) and calving difficulty (CD), the majority beef population presents relatively higher heritability than dairy cattle. Compared to bovine, genetic studies for buffalo reproductive traits are limited for age at first calving and calving interval traits. Several quantitative trait loci (QTLs), candidate genes, and SNPs associated with bovine reproductive traits were screened and identified by candidate gene methods and/or GWASs. The IGF1 and LEP pathways in addition to non-coding RNAs are highlighted due to their crucial relevance with reproductive traits. The distribution of QTLs related to various traits showed a great differences. Few GWAS have been performed so far on buffalo age at first calving, calving interval, and days open traits. In addition, we summarized the GS studies on bovine and buffalo reproductive traits and compared the accuracy between different reports. Taken together, GWAS and candidate gene approaches can help to understand the molecular genetic mechanisms of complex traits. Recently, GS has been used extensively and can be performed on multiple traits to improve the accuracy of prediction even for traits with low heritability, and can be combined with multi-omics for further analysis.

## Introduction

Reproductive traits are economically important for sustainable food production, especially for monotocous livestock, such as cattle and buffalo. Low reproductive capacity or infertility can be described as an extended duration between two calvings. This problem requires additional inseminations, more veterinary attention, and hormonal treatments, which consequently alters the current and subsequent lactations (Boichard, 1990). Also, additional costs are raised due to culling and replacing animals with fertility problems (Roxström and Strandberg, 2002). Enhancing fertility is the best choice not only to reduce the culling cost but also to save important genetic materials and increase farm profit (Dekkers, 1991). Several countries have included female reproductive traits in the breeding goals to emphasize the genetic aspects of reducing fertility costs (FCOST) in dairy cattle (Kadarmideen and Simm, 2002). Herein, we emphasize the recent literature about genetic parameters, genome-wide association study (GWAS), and genomic selection (GS) for reproductive traits in cattle and buffalo over the past 20 years for researchers, who can integrate these traits in cattle and buffalo breeding programs and achieve optimum fertility.

In the previous study, reproductive traits were divided into binary, interval, and continuous traits with respect to statistical distribution (Berry and Evans, 2014). To better understand and utilize reproductive traits in livestock and breeding programs, they are reclassified as ovulation, mating, and calving-related traits from the physiological viewpoint (Cammack et al., 2009; Table 1).

**TABLE 1:** Physiological classification and description of reproductive traits.

**Trait category**	**Parameter**	**Description**
Ovulation	Ovulation rate	Corpus luteum (CL) number during mid-luteal phase of the estrous cycle
	Superovulation response	The biological potentiality of the cow in terms of total number of ova (TNO), transferable embryos (NTE), unfertilized ova (NUO) and degenerated embryos (NDE); total number of embryos (NE) and number of viable embryos (VE)
	Twinning rate	The proportion of cows giving birth to two or more calves in one pregnancy
Mating	Age of puberty (AOP)	Male: the age when a bull scrotal circumference reaches 26–29 cm (AGESC)*, or the age at which a bull first produces an ejaculate containing at least 50 million sperm with a minimum of 10% motility Female: the appearance of the first corpus luteum (AGECL), age at first behavioral estrus (AFO) or standardized age at first behavioral estrus (SFO) and plasma progesterone concentration
	Age at first calving (AFC)	The interval between the date of first calving and the date of birth of the cow
	Non-return rate (NRR)	The proportion of cows that are not subsequently rebred
	pregnancy rate (PR)	The percentage of cows to become pregnant
Calving	Calving interval (CI)	The period of time (days or months) between the birth of a calf and the birth of a subsequent calf, both from the same cow
	Days open (DO)	The period between calving and conception
	Calving difficulty (CD)	Dystocia, which is categorized into three degrees, including easy calving, slight problems, and difficult calving
	Length of production life (LPL)	Mainly focused on dairy cattle, length of service, tenure, etc. Such as fertility-/mastitis-/production-/determined PL (FPL/MPL/PPL)

*Most of the heritability studies for bulls’ puberty employed the AGESC 26–29 cm.

## Heritability Estimates of Reproductive Traits

Genetic variation, which is a variability in breeding values within a population for a trait under selection, significantly affects the accuracy of genetic selection. Heritability measures how much of the phenotypic variation is attributed to genetic variation, and affects the rate of genetic improvement for a trait over generations. Over the past 20 years, several studies were conducted to estimate the heritability of different reproductive traits in dairy cattle (Table 2), beef cattle (Table 3), and buffalo cows (Table 4).

**TABLE 2:** Heritability estimates of reproduction traits in dairy cattle.

**Category**	**Trait**	**Heritability**	**Breeds (Numbers/records)**	**References**
Ovulation	Superovulation responses	0.231 ± 0.091	Holstein (2,489)	König et al., 2007
		0.27 ± 0.08	Holstein (926)	Gaddis et al., 2017
		0.234 ± 0.046(CL) 0.159 ± 0.087(EM)	Holstein–Friesian (56)	Bényei et al., 2004
		0.15 ± 0.01	Holstein (150,971)	Jaton et al., 2020
		0.15 ± 0.01/0.17 ± 0.01(NE) 0.14 ± 0.01/0.14 ± 0.01(VE) (Log/Ans)	Holstein (137,446)	Jaton et al., 2016a
		0.145 ± 0.007/0.188 ± 0.033(NE) 0.136 ± 0.007/0.187 ± 0.034(VE) (*in vivo*/*vitro*)	Holstein (145661/5310 records) (*in vivo*/*vitro*)	Jaton et al., 2016b
	Twinning rate	0.11 ± 0.01(parity1) 0.16 ± 0.01(parity2) 0.14 ± 0.01(parity3)	Japanese Holsteins (1,323,946) (1053469) (750600)	Yutaka et al., 2015
		0.0192 ± 0.0009/0.142 ± 0.007 (LM/TLM)	Holsteins (658436 cows/1440540 records)	Lett and Kirkpatrick, 2018
		0.1	12 multiple breeds (9272 females)	Allan et al., 2007
		0.013(parity1) 0.022(parity2) 0.024(parity3) 0.026(parity4) 0.031(parity5)	Israeli Holstein (671,361) (460940) (304213) (188077) (104434)	Weller et al., 2008
Mating	Age of puberty	0.38	Friesian × Ethiopian Boran (399) Jersey × Ethiopian Boran (151)	Effa et al., 2011
	Age at first calving	0.4	Friesian × Ethiopian Boran (399) Jersey × Ethiopian Boran (151)	Effa et al., 2011
		0.26 ± 0.02	South African Holstein (20419)	Makgahlela et al., 2008
		0.20 ± 0.03/0.21 ± 0.03(uni-trait/bi-trait analysis)	Brazilian Girolando (10,900)	Canaza-Cayo et al., 2018
		0.219 ± 0.162	multiple dairy cows (224)	Ali et al., 2019
		0.17 ± 0.01 0.093 ± 0.037	Holstein–Friesian Other dairy breeds	Berry and Evans, 2014
		0.15 ± 0.04 (PM)/0.16 ± 0.04 (GPM)	7 breeds (9,106)	Konkruea et al., 2019
		0.111	Holstein (276,573)	Changhee et al., 2013
		0.103 ± 0.025	German Holstein heifers (721919)	Heise et al., 2017
		0.01 ± 0.07	Dairy Overo Colorado breed (2,043)	Montaldo et al., 2017
	Non-return rate	0.1292 (NRR45) 0.1460 (NRR90)	Holstein (21,405)	Ansari-Mahyari et al., 2019
		0.02 (Paternal NRR90) 0.02 (Maternal NRR90)	German Holstein (1193) (1283)	Kaupe et al., 2007
		0.012 (heifer NRR56) 0.015 (cow NRR56)	Holstein (2,527)	Müller et al., 2017
		0.011 ± 0.001(NRR56)	Holstein (386869)	Zhang et al., 2019
		0.027 ± 0.0004 0.020 ± 0.001	Holstein–Friesian Other dairy breeds	Berry et al., 2014
	Pregnancy rate	0.04/0.02/0.01 (DPR/CCR/HCR)	Holstein (2,107)	Gaddis et al., 2016
		0.04	Spanish Holstein (113375 records)	Gonzálezrecio and Alenda, 2005
Calving	Calving interval	0.17	Friesian × Ethiopian Boran (847) Jersey × Ethiopian Boran (559)	Effa et al., 2011
		0.16 ± 0.12 0.00 ± 0.09	Holstein (624) Swedish Red (460)	Tarekegn et al., 2019
		0.14 ± 0.211	multiple dairy cow (224)	Ali et al., 2019
		0.106 ± 0.015 (linear sire model) 0.103 ± 0.013 (linear animal model) 0.059 ± 0.006 (repeatability animal model)	Iranian Holstein (22,269)	Chegini et al., 2019a
		0.07 ± 0.013	Holstein (11674 records)	Toghiani, 2012
		0.044 ± 0.01	Holstein (20544)	Chegini et al., 2019b
		0.04 ± 0.003	Iranian Holstein (129199)	Hossein Salimi et al., 2017
		0.04	Spanish Holstein (96346 records)	Gonzálezrecio and Alenda, 2005
		0.034 ± 0.001 0.029 ± 0.004	Holstein–Friesian Other dairy breeds	Berry et al., 2014
		0.002 ± 0.02	Dairy Overo Colorado breed (3,488)	Montaldo et al., 2017
		0.01 ± 0.02 (CI_1_) 0.00 ± 0.04 (CI_2_) 0.08 ± 0.07 (CI_3_)	Brazilian Girolando (5327) (3444) (2229)	Canaza-Cayo et al., 2018
		0.03 ± 0.01(CI_1_) 0.04 ± 0.01(CI_2_) 0.04 ± 0.01(CI_3_) 0.03 ± 0.01(CI_4_)	South African Holstein (20419) (18589) (10681) (15529)	Makgahlela et al., 2008
		0.088 (CI_1_) 0.142(CI_2_)	Holstein (167996 records) (128080 records)	Changhee et al., 2013
	Days open/calving to conception interval	0.102	Canadian Holstein (3,729)	Nayeri et al., 2016
		0.09 ± 0.121	multiple dairy cows (224)	Ali et al., 2019
		0.06 ± 0.03	Holstein (3,682)	Saowaphak et al., 2017
		0.06 ± 0.008	Holstein (15895)	Toghiani, 2012
		0.04	Spanish Holstein (113375 records)	Gonzálezrecio and Alenda, 2005
		0.04 ± 0.003	Iranian Holstein (129199)	Hossein Salimi et al., 2017
		0.033/0.024 (Model1/2)	Korean Holstein (14,188)	Lee and Han, 2004
		0.026	Holstein (2,527)	Müller et al., 2017
		0.038 ± 0.002 0.030 ± 0.001	Holstein–Friesian Other dairy breeds	Berry et al., 2014
	Calving difficulty	0.132 ± 0.003	Holstein (734)	Maryam et al., 2016
		0.121 ± 0.024 (LM) 0.074 ± 0.012 (TM)	Walloon Holstein	Vanderick et al., 2015
		0.05 (paternal CE) 0.05 (maternal CE)	German Holstein (1267) (1287)	Kaupe et al., 2007
		0.048 (paternal CE) 0.039 (maternal CE)	Holstein (2,527)	Müller et al., 2017
		0.043 ± 0.0031/0.010 ± 0.0016 (LM1) 0.041 ± 0.0030/0.010 ± 0.0015 (LM2) 0.046 ± 0.0032/0.011 ± 0.0016 (LM3) 0.086 ± 0.0091/0.023 ± 0.0037 (TM) (direct/maternal CE)	Portuguese dairy cattle (320,953 records)	Silvestre et al., 2019
		0.02 ± 0.002	Iranian Holstein (132831)	Hossein Salimi et al., 2017
		0.015/0.030 (Model1/2)	Korean Holstein (14,188)	Lee and Han, 2004
	Length of productive life	0.16	German Holstein (1,286)	Kaupe et al., 2007
		0.12	Pinzgau Cattle	Egger-Danner et al., 2005
		0.102	Holstein (276,573)	Changhee et al., 2013
		0.10 ± 0.03	Holstein (4,739)	Saowaphak et al., 2017
		0.06/0.10/0.18/0.25 (LPL/FPL/MPL/PPL)	Swedish Red and White dairy cattle (538783)	Roxström and Strandberg, 2002
		0.04	Hungarian Holstein (1403747)	van der Linde et al., 2006

**TABLE 3:** Heritability estimates of reproduction traits in beef cattle.

**Category**	**Trait**	**Heritability**	**Breeds (Numbers/Records)**	**References**
Ovulation	Ovulation rate	0.12	MARC twinning herd (16,035)	Allan et al., 2014
		0.08	MARC 12 breeds of cattle (29485 records)	Allan et al., 2007
		0.02	multiple breeds	Piper et al., 2017
	Superovulation responses (VE)	0.56–0.65 (1 flush) 0.20–0.26 (3 flushes)	Nellore (405) (858)	Peixoto et al., 2004
	Twinning	0.1	MARC twinning herd (16,035)	Allan et al., 2014
		0.1	MARC 12 breeds of cattle (9272 records)	Allan et al., 2007
		0.062 ± 0.093 (RThM) 0.014 ± 0.018 (RLM)	Maremmana cattle (1,260)	Moioli et al., 2017
Mating	Age of puberty	0.31 ± 0.05 (AFO) 0.27 ± 0.04 (SFO) 0.56 ± 0.11 (AGECL) 0.78 ± 0.10 (AGE26)	Angus cattle (1513 records) (1588 records) Brahman heifers (1007) Brahman bulls (1118)	Morris et al., 2000 Fortes et al., 2012
		0.57 ± 0.12 0.52 ± 0.12 (AGECL)	Brahman heifers (1007) Tropical Composite heifers (1108)	Johnston et al., 2009
		0.35/0.22/0.11 0.22/0.33 0.24/0.32 (AGECL)	Brahman (397/371/206) Santa Gertrudis (1022/776) Droughtmaster (222/688)	Engle et al., 2019
		0.42–0.44	Nelore cattle (12964)	Forni and Albuquerque, 2005
		0.26 ± 0.03	Heifer Angus (629)	Morris et al., 2011
		0.221 ± 0.08 (univariate) 0.198 ± 0.06 (multivariate)	50% Red Angus, 25%Charolais and 25%Tarentaise (890)	Toghiani et al., 2017
		0.310 ± 0.050 (AFO)	Beef cattle	Berry and Evans, 2014
		0.16–0.20	1828 Beef CRC (868 Brahman and 960 Tropical Composite) 3695 SMF (979 Brahman,1802 Santa Gertrudis and 914 Droughtmaster)	Warburton et al., 2020
	Scrotal circumference	0.37 ± 0.06(SC-8 month) 0.44 ± 0.06 (SC-10 month) 0.42 ± 0.06 (SC-12 month)	Angus cattle (1702 records) (1691 records) (1671 records)	Morris et al., 2000
		0.48 ± 0.02 (AGE365) 0.52 ± 0.02 (AGE450)	Brazilian Nellore (27567 records)	Kluska et al., 2018
		0.397 ± 0.011 (AGE365)	Nelore (135862 records)	Schmidt et al., 2019
		0.33 ± 0.07 (AGE365) 0.41 ± 0.07 (AGE450)	Guzera beef cattle (1773) Guzera beef cattle (2091)	Tramonte et al., 2019
		0.29 (AGE365)	Nelore cattle (66986 records)	Costa et al., 2020
		0.18 ± 0.02 (AGE365)	Charolais, Charbray, and Charolais-Zebu crosses (18,972)	Martínez-Velázquez et al., 2020
	Age at first calving	0.31 ± 0.016	Crossbred *Bos taurus* (64380 records)	Berry et al., 2014
		0.27 ± 0.12	Asturiana de los Valles (1226 records)	Goyache and Gutiérrez, 2001
		0.24 ± 0.04	Brazilian Nelore cattle (762)	Mota et al., 2017
		0.235 ± 0.018	Asturiana de los Valles (2533 records)	Gutiérrez et al., 2002
		0.220 ± 0.11	Jersey × Red Sindhi (313)	Vinothraj et al., 2016
		0.215 ± 0.026	Japanese Black Cows (24595 records)	Oyama et al., 2002
		0.20	Nelore cattle (1853)	Costa et al., 2019
		0.20–0.22	Simmental (3,063)	Amaya-Martínez et al., 2020
		0.17 ± 0.04	Brahman-Angus (909)	Elzo et al., 2018
		0.158 ± 0.039	Japanese Black cows (2,078)	Setiaji and Oikawa, 2019
		0.137 ± 0.008	beef cattle	Berry et al., 2014
		0.13 ± 0.130	Crossbred heifers (538 records)	Akanno et al., 2015
		0.11 ± 0.01	Brazilian Nellore (18526 records)	Kluska et al., 2018
		0.10 ± 0.01 (multi-trait model) 0.08 ± 0.01 (single-trait model)	Hanwoo cows (15,355)	Lopez et al., 2019
		0.10 ± 0.01	Nelore beef cattle (25,594)	Boligon and Albuquerque, 2011
		0.20/0.19/0.18/0.09 (LM/SM/PM/TLcens)	Brazilian Brahman cattle (53703 records)	Lázaro et al., 2019
		0.08	Nelore cattle (374665 records)	Costa et al., 2020
		0.06/0–0.15 0.13/0.06–0.13 (AMl/MHNRHOP1)	Limousine (18,500) Charolais (4,330)	de Rezende et al., 2020
		0.06–0.08	Nelore cattle (18615)	Forni and Albuquerque, 2005
		0.039 ± 0.039 (univariate) 0.031 ± 0.01 (multivariate)	50% Red Angus, 25%Charolais and 25%Tarentaise (1117)	Toghiani et al., 2017
	Non-return rate	0.020 ± 0.029 (1st parity) 0.014 ± 0.022 (2nd parity) 0.013 ± 0.034 (3rd parity) 0.013 ± 0.017 (repeatability model)	Japanese Black cows (2,078)	Setiaji and Oikawa, 2019
	Pregnancy rate	0.21 ± 0.009	Angus (1,299)	Doyle et al., 2000
		0.14 ± 0.099	Crossbred heifers (734 records)	Akanno et al., 2015
		0.12 ± 0.05 (yearlings) 0.08 ± 0.064 (2-year-olds)	Angus cattle (1190 records) (711 records)	Morris et al., 2000
		0.027 ± 0.38 (1st parity) 0.023 ± 0.034 (2nd parity) 0.021 ± 0.036 (3rd parity) 0.022 ± 0.007 (repeatability model)	Japanese Black cows (2,078)	Setiaji and Oikawa, 2019
		0.025/0.014/0.023/0.014 (model 1/2/3/4)	Sistani beef cattle (1489 records)	Faraji-Arough and Rokouei, 2016
Calving	Calving interval	0.222 ± 0.101	Jersey × Red Sindhi (522)	Vinothraj et al., 2016
		0.125 ± 0.020	Asturiana de los Valles (2007 records)	Gutiérrez et al., 2002
		0.12 ± 0.03	Asturiana de los Valles (1851 records)	Goyache and Gutiérrez, 2001
		0.105 ± 0.008	Nelore (33735 records)	Schmidt et al., 2019
		0.09 ± 0.02 (CI_1_)	Brahman-Angus (447)	Elzo et al., 2018
		0.02 ± 0.02 (CI_1_) 0.02 ± 0.04 (CI_2_) 0.06 ± 0.03 (mean CI)	Nelore (2642) (1437) (2888)	do Amaral Grossi et al., 2016
		0.049 ± 0.048 (CI_1_) 0.043 ± 0.045 (CI_2_) 0.048 ± 0.042 (CI_3_) 0.047 ± 0.009 (repeatability model)	Japanese Black cows (2,078)	Setiaji and Oikawa, 2019
		0.047 ± 0.009	Japanese Black Cows (72740 records)	Oyama et al., 2002
		0.032 ± 0.004	beef cattle	Berry et al., 2014
		0.056/0.040/0.033/0.032 (model 1/2/3/4)	Sistani beef cattle (1489 records)	Faraji-Arough and Rokouei, 2016
		0.01 ± 0.05 (CI_1_) 0.04 ± 0.02 (CI_2_) 0.07 ± 0.03 (CI_3_) 0.03 ± 0.01 (multi-trait model)	Hanwoo cows (1936) (11144) (8201) (32599)	Lopez et al., 2019
		0.02 ± 0.004	Crossbred *Bos taurus* (101864 records)	Berry and Evans, 2014
	Days open/calving to conception interval	0.192 (model 1) 0.091 (model 2) 0.168/0.197/0.170/0.091 (model3) 0.154/0.132 (model4) 0.135/0.090/0.086 (model5)	Asturiana de los Valles (21349 records) (3250/3416/13783/900 records) (6666/14683 records) (21349 records)	Goyache et al., 2005
		0.110 ± 0.04	beef cattle	Berry et al., 2014
		0.110 ± 0.04	Angus (1680 records)	Morris et al., 2000
		0.09/0.045/0.096/0.049 (model 1/2/3/4)	Sistani beef cattle (1489 records)	Faraji-Arough and Rokouei, 2016
		0.047 ± 0.009	Japanese Black cows (72740 records)	Oyama et al., 2002
		0.042 ± 0.044 (1st parity) 0.034 ± 0.052 (2nd parity) 0.034 ± 0.033 (3rd parity) 0.036 ± 0.021 (repeatability model)	Japanese Black cows (2,078)	Setiaji and Oikawa, 2019
		0.02 ± 0.05 (1st parity) 0.09 ± 0.02 (2nd parity) 0.08 ± 0.03 (3rd parity) 0.03 ± 0.01(multi-trait model)	Hanwoo cows (1726) (7308) (5888) (32465)	Lopez et al., 2019
	Calving difficulty	0.42	Asturiana de los Valles (7298 records)	Goyache and Gutiérrez, 2001
		0.325 ± 0.022	Asturiana de los Valles (35,395 records)	Cervantes et al., 2010
		0.32 ± 0.174	Crossbred heifers (543 records)	Akanno et al., 2015
		0.29 ± 0.10	multi breeds (5,795)	Ahlberg, 2014
		0.250 ± 0.018	Crossbred *Bos taurus* (100445 records)	Berry and Evans, 2014
	Length of productive life	0.096 ± 0.001	Multiple breeds (21,895)	Brzáková et al., 2019

**TABLE 4:** Heritability estimates of reproduction traits in buffalo.

**Trait**	**Heritability**	**Breeds (Numbers/records)**	**References**
Age at first calving	0.28 ± 0.03	Murrah buffalo (827)	Kumar et al., 2015
	0.226 ± 0.154 0.16	Surti buffalo (48) Murrah water buffalo (2290 records)	Rathod et al., 2018 de Araujo Neto et al., 2020
	0.16 ± 0.04	Murrah buffalo (2389 records)	Barros et al., 2016
	0.16 ± 0.12	Murrah buffalo (167)	Thiruvenkadan et al., 2010
	0.17 ± 0.02	Murrah buffaloes (3,431 records)	Camargo et al., 2015
	0.135 ± 0.035	Indian Murrah buffalo (1,456 records)	Gupta et al., 2015
	0.11 ± 0.06	Egyptian buffalo (1911 records)	El-Bramony, 2011
	0.07 ± 0.05	Murrah buffalo (1,578)	Seno et al., 2010
calving interval	0.55 ± 0.131	Surti buffalo (158)	Rathod et al., 2018
	0.234 ± 0.175	Indian Murrah buffalo (1,456 records)	Gupta et al., 2015
	0.14 ± 0.07 (CI_1_)	Murrah buffalo (1,578)	Seno et al., 2010
	0.09 ± 0.13	Murrah buffalo (506)	Thiruvenkadan et al., 2010
	0.085 ± 0.134	Iranian Khuzestan buffalo (146 records)	Morammazi et al., 2007
	0.07 ± 0.05	Egyptian buffalo (1911 records)	El-Bramony, 2011
	0.06 ± 0.01	Egyptian buffalo (2,066)	El-Bramony and Reclamation, 2014
	0.06 ± 0.01	Murrah buffaloes (4729 records)	Camargo et al., 2015
	0.05 ± 0.08	Mehsana buffalo (812 records)	Galsar et al., 2016
	0.05 ± 0.01	Murrah buffalo (5672 records)	Barros et al., 2016
	0.03(CI_1_)	Murrah water buffalo (765 records)	de Araujo Neto et al., 2020
Days open	0.14 ± 0.03	Murrah buffaloes (6894 records)	Camargo et al., 2015
Calving difficulty	0.16/0.19/0.06/0.08/0.09/0.04/0.11 (parity1–7)	Iraqi Buffalo (360)	Al-Khuzai et al., 2019

In dairy cattle, all ovulation-related traits range from low to moderate heritabilities (Table 2). The heritability estimate of the superovulation response was about 0.15 in Holstein cows (Jaton et al., 2020). Regarding mating-related traits, heritability estimates for age of puberty (AOP) and age at first calving (AFC) were moderate in most cattle populations, except for AFC in the Chile population (*h*^2^ = 0.01) (Montaldo et al., 2017). Likewise, the heritabilities of non-return rate (NRR) and pregnancy rate (PR) of Holstein dairy cows and Brown Swiss cattle were low (Gaddis et al., 2016; Tiezzi et al., 2018; Ansari-Mahyari et al., 2019; Zhang et al., 2019). Regarding the superovulation response and twinning rate, heritability was higher for superovulation, indicating a response to hormone treatment is more heritable than natural ovulation in dairy cows. Non-return and PR are directly related to reproductive outcomes. Unfortunately, the heritability estimates for these two traits were remarkably low. Besides, dairy cows’ calving-related traits, including calving interval, days open, calving difficulty (CD), and the length of the productive life, were all of low heritability. Therefore, management practices (reproductive management, balanced nutrition, etc.) and/or environmental factors are of significant importance for improving reproductive efficiency and preventing reproductive disorders in dairy cows. Thus, selection on dairy cows’ AOP, first calving, and superovulation response may gain more progression than other traits.

In beef cattle, the superovulation response had higher heritability than those of ovulation rate, and twinning rate was similar to those reported in dairy cattle (Table 3). Regarding mating-related traits, AOP had moderate to high heritability estimates in most beef populations; for example, the estimate reached 0.78 in the Brahman bull population (Fortes et al., 2012). The h^2^ for scrotal circumference was also reported to have moderate to high estimates. Excluding the Angus population (0.2) (Doyle et al., 2000) in beef cattle, the NRR and PR of heritability were low, as reported in dairy cattle. The heritabilities for calving difficulties in beef cattle had moderate to high estimates, unlike those reported in dairy cattle with low heritabilities. In comparison, other mating-related reproductive traits, such as DO, NRR, CI, and length of productive life had low heritabilities similar to dairy cattle. Taken together, selections on beef cow’s AOP, calving difficulties, DO, NRR, and CI traits may gain more progression due to the moderate to high estimates of heritabilities compared with other traits (Cassell, 2009).

The excellent milk quality and the limitation of buffalo milk yield contribute to the breeding selection focusing more on milk production traits in buffalo compared with reproductive traits. Currently, there are limited studies for estimating genetic parameters for reproductive traits in buffalo species, mainly for AFC and CI (Table 4). The heritability estimates of AFC in the buffalo population is close to Holstein cattle (Gupta et al., 2015; Kumar et al., 2015; Barros et al., 2016; Rathod et al., 2018). Most studies showed that the heritability of CI is low, mostly below 0.1 (Morammazi et al., 2007; Thiruvenkadan et al., 2010; El-Bramony and Reclamation, 2014; Camargo et al., 2015). However, the highest record for CI was 0.55 in Surti buffalo, which may be due to the limited numbers of lactation records and/or number of parities per sire monitored (Rathod et al., 2018). The heritabilities of DO (Camargo et al., 2015) and CD (Al-Khuzai et al., 2019) were similar to those reported in dairy cattle.

Comparing heritabilities between different traits in dairy and beef cattle along with buffalo, we found that:

(1) Most of the reproductive traits had low habitabilities, but not all. In the dairy and beef cattle, AOP showed high heritabilities. The heritability estimates for scrotal circumference of the beef bull were medium to high. Also, the superovulation response in dairy and beef cattle was worthy of notice. These moderate to high heritability traits could be applied to the selection and breeding system.
(2) The heritability estimates for calving intervals, NRR, days open, and length of reproductive life in most populations were very low, which indicated that these traits would be influenced and improved by proper management practices. The application of synchronization-timed AI protocol (Goodling et al., 2005), body composition control, reproductive disorder treatment, and culling on time would benefit the related performance.
(3) The heritability of the same trait varies greatly among different breeds. For instance, the heritability of age at first calving was as high as 0.4 in a crossbreed of dairy cows (Effa et al., 2011), while the Dairy Overo Colorado breed was as low as 0.01 (Montaldo et al., 2017). The heritability of CI reported in Surti buffalo is 0.55 (Rathod et al., 2018) compared to the Murrah buffalo cows near to 0.1 (Thiruvenkadan et al., 2010). Although heritability was estimated using paternal half-sib correlation methods in both studies, lactation records, number of buffaloes, and sired by bulls were higher for Murrah buffaloes. Even in the same breed, the different populations showed varied values, which may related to different management and performance.
(4) For most of the reproductive traits, beef cattle had higher heritability estimates compared to those estimated in dairy cattle for the AOP and CD (Tables 2, 3). Either the genetic makeup or the fact that dairy cows are more susceptible to reproductive diseases, such as endometritis, vaginitis, ovarian cyst, and mastitis, due to high energy consumption for milk production may be the reason for this difference.
(5) The breeding progress of buffalo is slow compared to dairy and beef cattle, as a few studies have reported during the last decade. Further large-scale studies are required to accurately estimate the genetic parameters for different reproductive traits in buffalo populations.

## Marker-Associated Studies for Bovine and Buffalo Reproductive Traits

Concerning the disadvantages of the long cycle and not up-to-mark efficiency of traditional breeding, several association analyses were performed to identify genomic loci associated with the trait variation to find the possible candidate genes or to detect causative mutations. This section summarized the GWAS and candidate gene studies for bovine and buffalo reproductive traits published in the past 20 years (2000–2020) (Supplementary Tables 1–3).

At present, there are few marker-assisted selection (MAS) studies on the reproductive traits of buffalo. In this regard, *FSHR*, *INHA*, *LHCGR*, and *OPN* were reported to have significant effects on the buffalo superovulation responses. So far, few GWAS have been performed on buffalo reproductive traits (Camargo et al., 2015; Li et al., 2018a, b; de Araujo Neto et al., 2020). Previous GWASs for reproductive traits (Camargo et al., 2015; Li et al., 2018a) were conducted using the bovine reference genome assembly, and the results are expressed for bovine autosomes (BTA). Camargo et al. (2015) reported some candidate genes (*TPCN1*, *SCG5*, and *Fig 4*) associated with reproductive traits such as AFC, CI, and DO in buffalo. Also, Li et al. (2018a; 2018b) found 25 SNPs in 13 genes related to reproductive traits by integrating RNA-seq and GWAS methods. They also described significant SNPs on BBU 6, 9, and 15 [corresponding to bovine chromosomes 3, 7, 14, and 8: equivalence presented by Cribiu et al. (2001)]. Recently, ssGBLUP was employed to identify genomic regions affecting AFC and first calving interval (FCI) in buffalo cows and select candidate loci and gene expression (de Araujo Neto et al., 2020). They reported that the observed candidate regions for both traits (CI, AFC; explaining a large proportion of variance for both traits) were located on BBU 3, 12, 21, and 22. Also, candidate regions were found on BBU 6, 7, 8, 9, and 15 for age at first calving and on BBU 4, 14, and 19 for FCI. The *ROCK2*, *PMVK*, *ADCY2*, *MAP2K6*, *BMP10*, and *GFPT1* genes are the main candidates for reproductive traits in water dairy buffaloes, which may be used in the future for animal breeding programs or for gene expression studies of the species (de Araujo Neto et al., 2020). The *GFPT1* and *BMP10* genes are interesting because they have been detected for both traits, which may be related to a possible pleiotropic effect.

The candidate gene studies for bovine reproductive traits mostly used genes of hormones and/or growth factors and their receptors as candidates (Tang et al., 2011; Yang et al., 2013; Arslan et al., 2017). For example, polymorphisms in the *GnRH*, *GnRHR*, *LEP*, and *LHCGR* were studied for association with reproductive traits of buffalo bulls. Notably, genes involved in IGF1 and LEP pathways were reported to affect multiple reproductive traits. For example, *IGF1* could affect a variety of ovulation- and mating-related traits. *LEP* and *LEPR* showed significant effects on both breeding- and calving-related traits. Moreover, long non-coding RNA and ribosomal RNA could be future research directions since non-coding RNAs (U6 spliceosomal RNA) were reported to affect reproductive traits (Fortes et al., 2013; Nascimento et al., 2016; Buzanskas et al., 2017). The combination of GWAS and other omics studies are becoming more useful, as they provide a broad space for exploring candidate gene functions and related mechanisms.

Further, we visualized the chromosomal distribution of quantitative trait loci (QTL) in cattle related to each reproductive trait using the Cattle Quantitative Trait Locus Database (Cattle QTLdb) (Hu et al., 2019) (Supplementary Figures 1–3). Only 11 QTL related to ovulation-related traits were identified, and four of these were located on chromosome 5, where the *IGF1* gene is placed (Miller et al., 1992) (Supplementary Figure 1). The QTL for mating-related traits were spread throughout different chromosomes (Supplementary Figure 1A). The most abundant chromosome is BTX with 10237 QTL (96.4%) related to puberty. BTA2 (21QTLs, 19.6%) and BTA14 (15 QTLs, 14.0%) had the most associated loci for AFC (Supplementary Figure 1B). Most of the QTL for NRR were located on BTA17 (233421 QTLs, 94.7%). However, QTL for PR-related were scattered (Supplementary Figure 2). About 37.1% of QTL related to calving interval were enriched in BTA25 (17.5%) and BTA29 (19.6%). Whereas, BTA 21 enriched the most QTLs (44.8%) related to CD, and BTA18 had 30.7% of QTL related to the length of productive life.

Undoubtedly, these significantly enriched chromosomes (BTX related to puberty, BTA related to NRR, and BTA related to CD) could be directions for future research. Moreover, certain areas that affect multiple traits of different species also deserve further attention. For example, McClure et al. (2010) found one SNP related to CD at 49.1 Mb of BTA 20 in Angus cattle (McClure et al., 2010), while Ke et al. (2014) reported SNP in a similar region in dairy cattle affecting age at first calving. The relationship between these highly enriched chromosomal regions and various traits is worthy of further investigation.

Based on morphological and behavioral criteria, the domestic Asian water buffalo has two types (Macgregor, 1941). The two types have different chromosome numbers: river buffalo (*Bubalus bubalis*, 2n = 50) and swamp buffalo (*Bubalus bubalis carabanesis*, 2n = 48) (Ulbrich and Fischer, 1966). In addition, the chromosomal karyotype of hybrid buffalo is more complicated. Although presenting different species, buffalo and bovine share highly homologous chromosomes banding, as well as gene mapping (Amaral et al., 2008; Michelizzi et al., 2010; Kale et al., 2014). It is also reported that river buffalo and bovine chromosomes can be matched arm for arm at the cytogenetic level (Williams et al., 2017; Du et al., 2019). Despite the complicated genomic background of buffalo, candidate genes or their chromosome locations identified for the bovine reproductive traits could be considered as a valuable reference for buffalo.

## Genomic Selection for Reproductive Traits in Bovine and Buffalo

Phenotypic records for a trait of individuals and their relatives are used to estimate breeding values by employing the best linear unbiased prediction (BLUP) to facilitate animal selection for economically important traits (Henderson, 1984). It is believed for genetic selection that information at the DNA level can quicken the genetic progression compared to phenotypic data alone. The sparse map of genetic markers can be used to detect QTL (Georges et al., 1995). Combining genetic marker information with BLUP (Fernando and Grossman, 1989) showed an increase in the genetic gain by 8–38% (Fernando and Grossman, 1989; Goddard, 1996). The effectiveness of sparse markers in outbreeding species was limited, as an establishment of linkage phase between a marker and QTL is necessary for every family in which the marker is to be used for selection (Meuwissen et al., 2001).

The total number of SNP estimated at millions and the advent of DNA Chip technology made genotyping of many animals for many of these markers feasible and cost-effective. However, a dense marker map improved precision for QTL mapping by traditional linkage analysis (Darvasi et al., 1993). Therefore, a search for a different approach to efficiently use all this marker information remained necessary.

Considering a denser marker map, not only could some markers be close to QTL but also, in linkage disequilibrium with it, it was anticipated that some markers could have a positive effect on the quantitative traits across all families and be used for selection without the need to establish a Linkage phase in each family. Close markers can also be combined into a haplotype. Chromosome bearing the rare marker haplotype is likely to be identical by descent and hence carry the same QTL allele. Meuwissen et al. (2001), estimated the effect of the quantitative trait of the small chromosome segment defined by the haplotype of the allele that they carry. They concluded that it’s possible to accurately estimate the breeding value of animals that have no phenotypic records by estimating a large number of haplotype effects. Using least squares, all haplotype effects could not be estimated simultaneously. Even when only the largest effects were included, they were overestimated and the accuracy of predicting breeding value was low. Methods that assumed prior distribution for the variance associated with each chromosome segment gave a more accurate prediction of breeding values even when the prior was not correct. Selection based on breeding values predicted from markers could substantially increase the rate of genetic gain in animals and plants, especially if combined with reproductive techniques to shorten the generation interval. Selection based on pedigree has played an important role in the selective breeding improvement in domestic animals.

Quantitative traits are usually affected by many genes and, consequently, the benefits from the MAS are limited by the proportion of the genetic variance explained by the QTL. Hence, it is warranted to utilize all the QTL affecting the traits in MAS. Nevertheless, a dense marker map defines a very large number of chromosome segments and so there will be many effects to be estimated, probably more than there are phenotypic data points from which to estimate them (Meuwissen et al., 2001).

With the emergence of high-density SNP chips, such as Illumina chips [BovineHD BeadChip SNP, BovineSNP50 chip, High-Density Bovine SNP chip (777K)] and Axiom^®^ Buffalo Genotyping Array (90K), GS methods are improving livestock genetic evaluation systems. They have the advantages of high accuracy, short interval between generations, and rapid genetic progress.

At present, GS has been applied in cattle on a large scale, but mainly focus on milk production and carcass traits (Silva et al., 2014; Weller et al., 2017). The GS studies on reproductive traits in dairy and beef cattle, including AFC, puberty, NRR, PR, days open, and CD, are listed on Table 5.

**TABLE 5:** A summary of genomic selection studies for reproductive traits.

**Traits studied**	**Breed (country)**	**Chip size**	**Validation population size**	**Models**	**Response variable**	**Accuracy of prediction**	**Regression coefficients**	**References**
Age at first calving	Nelore (Brazil)	Illumina BovineHD	1,853	GBLUP BAYESCπ IBLASSO	dEBV	0.38(GBLUP), 0.39(IBLASSO) 0.42(BAYESCπ)	0.88(GBLUP), 1.14(IBLASSO) 0.81(BAYESC)	Costa et al., 2019
	Nelore (Brazil)	Illumina Bovine 70 K	714	BayesA BayesB BayesCπ BLASSO BRR	dEBV	0.24(BayesA) 0.23(BayesB) 0.33(BayesCπ) 0.24(BLASSO) 0.38	0.62 0.63 0.65 0.83 0.65	Mota et al., 2018
	Nelore (Brazil)	Illumina BovineHD	2,241	BayesC	EBVs	0.64	0.9	Boddhireddy et al., 2014
	crossbred animals (Thai)	GeneSeek 80k chip	8,361	ss GBLUP ssGBLUPS1 ssGBLUPS2	EBV	0.297 0.298 0.264		Laodim et al., 2019
	Gyr dairy cattle (Brazil)	GeneSeek SGGP-20Ki Illumina BovineSNP50 GeneSeek GGP-75Ki Illumina BovineHD	422 bulls and 1582 cows	GBLUP	dEBVs	0.380	0.968/0.960 0.966/0.958 0.967/0.959 0.968/0.970 (bulls/bulls and cows)	Boison et al., 2017
	CGC: 50%Red Angus 25%Charolais 25%Tarentaise	BovineSNP50 chip	1117 records	BayesA BayesB BayesCπ	EBVs	0.148 0.143/0.154/0.146 (π = 0.99/0.95/0.90) 0.150		Toghiani et al., 2017
Scrotal circumference	Braford and Hereford (Brazil)	Illumina BovineSNP50K Illumina BovineHD	3680 (2997 Braford and 683 Hereford)	tsGBLUP/ssGBLUP	EBVs/dEBVs	0.28–0.33 0.15–0.17	0.50–1.10 0.55–1.13	Piccoli et al., 2020
	Brangus	GGP−LDV3 chip (1074) GGP−LDV4 chip (1535) Illumina BovineSNP50 (261) GGP−HDT (295) GGP−UHD (628) Illumina Bovine HD (4)	3,797	tsGBLUP ssGBLUP	EBVs/dEBVs	0.717 0.634		Lopes et al., 2018
	Nelore cattle (Brazil)	Illumina BovineHD (763) Illumina BovineSNP50 (1478)	2,241	BayesC	EBVs	0.59/0.59 (AGE365/450) 0.57/0.56 (AGE365/450)	0.95/0.93 (AGE365/450) 0.89/0.86 (AGE365/450)	Boddhireddy et al., 2014
	Nelore bulls (Brazil)	Illumina BovineHD	691	GBLUP Bayes C BLASSO	dEBV	0.68(GBLUP0) 0.71(GBLUP20) 0.72(Bayes C) 0.72(BLASSO)	1.27 (GBLUP0) 1.44(GBLUP2) 1.68(BAYESC) 1.65(BLASSO)	Neves et al., 2014
	Angus’ sires (America)	Illumina BovineSNP50	439	BayesC	dEBVs	0.487 (K-means)/0.600 (Random)	0.916 (K-means)/ 0.983 (Random)	Saatchi et al., 2011
Puberty (age at first corpus luteum)	Beef CRC: (882 Brahman and 990 Tropical Composite) Smart Futures: (974 Brahman, 1798 Santa Gertrudis, and 910 Droughtmaster)	Illumina BovineSNP50 chip GeneSeek GGP-LD array	1,872 3682	GBLUP	EBVs	0.49 ± 0.06 (Tropical Composite) 0.52 ± 0.07 (Brahman) (80% CRC + SF)		Engle et al., 2019
	50%Red Angus 25%Charolais 25%Tarentaise	BovineSNP50 chip	890	BayesA BayesB BayesC	EBVs	0.237 0.188/0.235/0.242 (π = 0.99/0.95/0.90) 0.226		Toghiani et al., 2017
	CRC(2174) and Validation cows (4286)	Illumina BovineHD Illumina 7K Illumina BovineSNP50K	6,460	GBLUP	EBVs	0.33 (Brahman) 0.15 (Tropical Composite)		Zhang et al., 2014
Non-return rate	Holstein (Canada)	Illumina Bovine SNP50	317 (first) and 489 (later)	ssGBLUP msGBLUP	GEBV DGV	0.39/0.33 (first/later)	0.63–0.97 (first) 0.81–1.35 (later)	Guarini et al., 2018
Heifer pregnancy rate	Angus sires (America)	Illumina BovineSNP50	133	BayesC	dEBVs	0.269 (K-means)/0.378 (Random)	1.337 (K-means)/1.580 (Random)	Saatchi et al., 2011
	Nelore (Brazil)	Illumina BovineHD (763) Illumina BovineSNP50 (1478)	2,241	BayesC	EBVs	0.64 0.64	0.89 0.87	Boddhireddy et al., 2014
Days open	Holstein (North America)	Illumina Bovine SNP 50 TM Chip	6,515	GBLUP	dEBV	0.50	0.9	Forutan et al., 2018
Calving ease direct/maternal (CED/CEM)	Brangus (CED/CEM)	GGP−LDV3 chip (1074) GGP−LDV4 chip (1535) Illumina BovineSNP50 (261) GGP−HDT (295) GGP−UHD (628) Illumina Bovine HD (4)	3,797	tsGBLUP ssGBLUP	EBVs dEBVs	0.451/0.512 0.337/0.266 (CED/CEM)		Lopes et al., 2018
	Holstein (Canada) (calving ease)	Illumina Bovine SNP50	438 (first) and 363 (later)	ssGBLUP msGBLUP	GEBV DGV	0.76/0.69 (first/later)	0.71–1.09 (first) 0.56–0.82 (later)	Guarini et al., 2018
	Angus bulls (America) (CED/CEM)	Illumina BovineSNP50 BeadChip	3180	BayesC	dEBVs	CED:0.488/0.617 CEM:0.416/0.571 (K-means/Random)	CED:0.942/1.007 CEM:1.181/1.277 (K-means/Random)	Saatchi et al., 2011
	Norwegian Red bulls (calving ease)	Affymetrix 25K MIP-SNP chip	500	GBLUP BayesB MIXTURE	GW-EBV	0.406/0.382 0.411/0.392 0.429/0.401 (Cohort//Random masking)	1.192/1.104 0.932/0.953 0.998/0.862 (Cohort//Random masking)	Luan et al., 2009

For AFC, the accuracy of genomic prediction was varied among different populations and methods. In the Nellore breed, the accuracy of prediction for AFC was 0.64 (Boddhireddy et al., 2014); however, another scholarly journal reported that the accuracy ranged between 0.38 and 0.42 by three different models (Costa et al., 2019). The prediction accuracy is around 0.23–0.33 in another Nellore cow population (Mota et al., 2018). Using the ssGBLUP model, the accuracy of prediction for AFC was 0.299 in the Thai native breed (Laodim et al., 2019), and was 0.56 in the Gyr dairy cattle breed (Boison et al., 2017).

Genomic selection studies on puberty (scrotal circumference and age at first corpus luteum) showed that the accuracy performance of different models is above 0.6 (Boddhireddy et al., 2014; Neves et al., 2014; Toghiani et al., 2017; Lopes et al., 2018; Engle et al., 2019). However, the accuracy was decreased dramatically in crossbred populations (Zhang et al., 2014; Piccoli et al., 2020). The limited reference population in the hybrid population and the general traits of the reference population have no direct counterpart in the validation population, which may be the reason for this decrease.

In the PR studies, the accuracy of prediction was 0.269 in the Angus population (Saatchi et al., 2011) and 0.64 in Nelore cattle (Boddhireddy et al., 2014). For CD, the highest accuracy was 0.516 in Brangus using GBLUP models (Lopes et al., 2018), and the prediction accuracy of different beef cattle breeds is around 0.45 among different models (Luan et al., 2009; Saatchi et al., 2011), while the accuracy in dairy cows was lower by 0.24–0.34 (Guarini et al., 2018).

Regarding buffalo studies, genomic evaluation reports are very limited either for productive or reproductive traits. There is only one published study for AFC and CI in buffalo (de Araujo Neto et al., 2020). Genomic evaluation studies in buffalo are still in the developing stage. The main limitation of applying genomic evaluation in buffalo is the lack of a well-structured reference population. Since the number of individuals with both genotypic and phenotypic information in each country is still limited, a multi-breed genomic evaluation would be the best alternative (Liu et al., 2018; Abdel-Shafy et al., 2020a, b).

## Conclusion and Perspectives

Reproductive traits were depreciated during selection indexes to improve the genetic potential of livestock. Hence, the recently desired gains are being practiced to ensure that the all TMI (total merit index) traits show a positive response or, at the very least, no negative response. However, the statistical data from the Council on Dairy Cattle Breeding (CDCB)^1^ indicated that, without severely slowing genetic gain for milk production, the daughter PR has stabilized and the declining trend has been reversing since 2003. A similar trend has also been demonstrated by García-Ruiz et al. (2016). Moreover, several pregnancy-related SNPs with neutral associations with milk production in Holstein bulls were identified (Cochran et al., 2013). It elicits the possibility of increasing fertility without reducing productive performance during selection.

Unlike dairy and beef cattle, few studies have been performed so far for reproductive traits in buffalo. Methods such as GWAS and GS require a large group size, well-structured pedigree, and accurate phenotypic records, which are big challenges for buffalo populations. The first reference for buffalo genome sequencing was released in 2017 (Williams et al., 2017), lacking the sequence in the chromosome and genes annotation, which was completed and updated in 2019 (Low et al., 2019; Mintoo et al., 2019). It will quicken the GS research and be significantly helpful in promoting buffalo breeding.

Dissimilar to dairy production traits, GWAS for reproductive traits seems to be underpowered and has difficulty in finding major QTL. It still provides genetic variability across many genome-wide genes and intragenic regions for complex trait studies, which greatly increases the understanding of complex traits’ molecular genetic mechanisms.

For reproductive traits with low heritability, the genetic gain using GS is improved three to four times per year compared to traditional methods (García-Ruiz et al., 2016). However, GS is also facing some difficulties, especially for buffalo, such as lacking an optimum population structure with record and some species having no dense marker maps yet. Its accuracy is limited by the reference population’s size and SNP marker density, which is obvious in some hybrid populations. In developing countries, there is a lack of complete historical records, and the number of genotyped animals has limited the development of GS. Also, for those traits with low to high heritability (such as puberty, age at first calving, and CD), multivariate GS can performed on multiple traits to improve prediction accuracy. In addition, multi-breed genomic evaluation can be used for populations with limited size. Besides, multi-omics data integration and analysis are gaining more attention from fields such as genomics, transcriptomics, and epigenomics.

## Author Contributions

GH contributed to the conception and design of the study. BS wrote the first draft of the manuscript and collected the data. CD, HS, MA, and YY wrote sections of the manuscript. NG, HA, SM, YZ, TD, LY, and SZ revised the manuscript and made profound suggestions. All authors contributed to manuscript revision and read and approved the submitted version.

## Conflict of Interest

The authors declare that the research was conducted in the absence of any commercial or financial relationships that could be construed as a potential conflict of interest.

## Footnotes

1 https://queries.uscdcb.com/eval/summary/trend.cfm
