# Supplementary material for: Genetic Features of Reproductive Traits in Bovine and Buffalo: Lessons From Bovine to Buffalo
Source: Front Genet. 2021 Mar 23;12:617128. doi: 10.3389/fgene.2021.617128 (PMC8021858; doi:10.3389/fgene.2021.617128)
Supplement: Supplementary file 1 [file Data_Sheet_1.docx]

| **Supplement Table1** **Quantitative** **trait** **loci** **(QTLs)** **and** **candidate** **genes** **associated** **with** **bovine and buffalo** **ovulation-related** **traits** | | | | |
| --- | --- | --- | --- | --- |
| **Trait** | **Method** | **Breed** | **located** **chromosomes (QTL/genes)** | **Reference** |
| **Ovulation** **rate** | GWAS | MARC twinning herd, Angus (40), Hereford (23), Holstein (53), Jersey (48) and Simmental (24) | BTA10 (SMAD3, SMAD6, IQCH) | Kirkpatrick and Morris (2015) |
|  |  | MARC twinning herd | BTA5 | Allan et al. (2014) |
|  |  | Japanese Black cattle (639) | BTA7 (GRIA1) | Mayumi et al. (2010) |
|  |  | MARC twinning herd | BTA7, 14, 19 | Gonda et al. (2004) |
|  |  | MARC twinning herd (307) | BTA5, 7, 10, 19 | Arias and Kirkpatrick (2004) |
|  |  | MARC twinning herd | BTA5, 7, 10, 19 | Kirkpatrick et al. (2000) |
|  |  | MARC twinning herd (181) | BTA5, 7, 23 | Kappes et al. (2000) |
|  |  | Norwegian cattle (285) | BTA5, 7, 12, 23 (IGF1) | Lien et al. (2000) |
|  |  | MARC twinning herd (approximately 750) | BTA7, 23 | Blattman et al. (1996) |
|  | Candidate genes | Brahman (932) and Tropical Composites (1097) | CAPN1 | Collis et al. (2012) |
| **Superovulation** **responses** | GWAS | Holstein (59,586) | BTA2, 11, 25, 29 (PADI2, NDUFA8, MORN5, PTGS1, OR1L8, DENND1A, CRB2, RALGPS1, MAD1L1, PANX1, POLA2) | Jaton et al. (2018) |
|  |  | Holstein (13,643) | BTA5, 8, 13,​​ 14, 16, 21(NPR2, TLE4, FZD6, CTHRC1, DHRS7, TOX, PARP1, KIAA1462, ZNF438, MTPAP, MUSK, MAP3K8, NSMAF, SDCBP, CA8, RAB2A, CHD7, SIX6, SVIL, LRRC9, PCNXL4, PP2C-α, LOC101902937, SEC14L1, TGFBR1) | Gaddis et al. (2017) |
|  | Candidate genes | Holstein (264) | LHCGR | Arslan et al. (2017) |
|  |  | Buffalo (45) | FSHR, INHA, LHCGR, OPN | Yang et al. (2016) |
|  |  | Holstein (171) | INHBA | Yang et al. (2014) |
|  |  | Holstein (43) | FSHR | Cory et al. (2013) |
|  |  | Holstein (171) | GDF9 | Tang et al. (2013) |
|  |  | Holstein (171) | IGF1R | Yang et al. (2013) |
|  |  | Nelore cattle (217) | GDF9, FGF8, BMRP2, LHCGR | Santos-Biase et al. (2012) |
|  |  | Holstein (171) | LHCGR | Tang *et al.* (2013) |
|  |  | Holstein (127) | LHCGR | Yu et al. (2012) |
|  |  | Holstein (118) | INHA | Tang et al. (2011) |
|  |  | Holstein (171) | PGR | Yang et al. (2011) |
|  |  | Holstein (171) | FSHR | Yang et al. (2010) |
| **Twinning** **trait** | GWAS | MARC twinning herd | BTA5 | Allan *et al.* (2014) |
|  |  | Holsteins (921) | BTA14 | Bierman et al. (2010a) |
|  |  | Holsteins (921) | BTA1, 2, 3, 6, 9, 22,23, 26 | Bierman et al. (2010b) |
|  |  | Holstein (233) | BTA2, 5, 14 (IGF1) | Kim et al. (2009b) |
|  |  | Holstein (671,361) | BTA1, 6, 7, 8, 14, 15, 23 (EMR1, CYP21) | Weller et al. (2008) |
|  |  | Holstein (1.3 million record) | BTA8, 10, 14, 21, 29 | Cobanoglu et al. (2005) |
|  |  | Holstein (586) | BTA5, 7, 19, 23 (PRNP) | Cruickshank et al. (2004) |
|  |  | Norwegian cattle (285) | BTA5 (CSSM22 - ILSTS66) | Meuwissen et al. (2002) |
|  |  | Norwegian cattle (285) | BTA5, 7, 19, 23 | Komisarek and Dorynek (2002) |
|  |  | Norwegian cattle (285) | BTA5, 7, 12, 2 3(CYP21, IGF1) | Lien *et al.* (2000) |
|  | Candidate genes | Luxi cattle (68) | GDF9, BMP15, FSHB, FSHR | Wei et al. (2015) |
|  |  | Maremmana breed (98) | GDF9, BMP15, BMPR1B | Marchitelli and Nardone (2015) |
|  |  | Tianzhu white yak (104) | FSH, FSHR | Xing et al. (2003) |
|  |  | Holstein (358) | IGF1 | Kim et al. (2009a) |
|  |  | Luxi cattle (306), Simmental (59), Angus (47), Simmental crossbreed (52) | RXRG | Huang et al. (2008a) |
|  |  | MARC twinning herd | SPP1 | Allan et al. (2007) |

| **Supplement Table 2 Quantitative** **trait** **loci** **(QTLs)** **and** **genes** **associated** **with** **bovine and buffalo mating-related** **traits** | | | | |
| --- | --- | --- | --- | --- |
| **Trait** | **Method** | **Breed** | **located** **chromosomes (QTLs/genes)** | **Reference** |
| **Age** **of** **puberty** | GWAS | Brahman cattle (1,007) | TSC22D2, KLF7, ARHGAP29, 7SK, MAP3K5, TLE3, TAF3, WDR5, TMEM68, PPP1R15B, NR2F2, GALR1, SUFU, KCNU1 | Melo et al. (2018) |
|  |  | Canchim (392) | BTA5, 13, 14, 20, 28 (SMIM23, PAPD7, ICE1, LOC101907249, EDARADD, RAP1B, SRGAP1, FOXM1, TOP1, STAU2, PEX2, FABP5, FABP12, MED30, TRHR) | Buzanskas et al. (2017) |
|  |  | Chinese Holstein (4,555) and Nordic Holstein (7,048) | BTA10, 13, 23 | Liu et al. (2017) |
|  |  | Nellore (55) | BTA 5, 6, 9, 10, 22 (U6, RELL1, LMBRD1, LRRFIP2) | Nascimento et al. (2016) |
|  |  | Tropical Composite (1085) | BTA2, 5, 14, 17, X (INHBC, INHBE, HELB, PLAG1, IGF1) | Fortes et al. (2013) |
|  |  | Brangus (~800) | BTA3, 8, 15, 16, 19, 24, 26, 27, 29 and X | Peters et al. (2013) |
|  |  | Brahman (843) and Tropical Composite (866) | BTA14, 15 | Hawken et al. (2012) |
|  |  | Jersey and Limousin (783) | BTA21 (TEXAN10 ) | Morris et al. (2009) |
|  | Candidate genes | Sahiwal and Frieswal (250) | OAS1 | Alex et al. (2018a) |
|  |  | Sahiwal and Frieswal (250) | OAS1 | Alex et al. (2018b) |
|  |  | Guzerat (159) | TG | Fernandez et al. (2017) |
|  |  | Nellore (385) | JY1 | Camargo et al. (2014) |
|  |  | Brahman (932) and Tropical Composites (1097) | DGAT1 | Collis *et al.* (2012) |
|  |  | Tropical Composite (866) and Brahman (843) | IGF1R, IGFBP2, IGFBP4, PERK (HUGO symbol EIF2AK3), PIK3R1, GSK3B and IRS1 | Fortes et al. (2012) |
|  |  | Angus (276) | IGF1, GNRHR | Lirón et al. (2012) |
|  |  | Holstein (509) | LEPR, NPY1 | Clempson et al. (2011a) |
|  |  | Nellore (100) | LEP, NPY | Vaiciunas et al. (2008) |
| **Age** **at** **first** **calving** | GWAS | Murrah water buffaloes (2290) | BBU 3, 6, 7, 8, 9,12,15, 21 and 15 (KCNJ16, KCNJ2, LOC102401240; MSTO1, LOC112585799, DAP3, LOC112585801, ASH1L, RUSC1, FDPS, LOC112585800, PKLR, HCN3, CLK2, SCAMP3, FAM189B, GBA, MTX1, THBS3, LOC112585544, MUC1, TRIM46, KRTCAP2, DPM3, SLC50A1, EFNA1, EFNA3, EFNA4, ADAM15, DCST1, DCST2, ZBTB7B, LENEP, FLAD1, CKS1B, SHC1, PBXIP1, LOC112585868, PMVK, LOC112585844, KCNN3; OCIAD2, OCIAD1, LOC112586198, LOC102398046, FRYL, ZAR1, SLC10A4, LOC112586199, SLAIN2, TEC, TRNAW-CCA, TXK, LOC112586200, NIPAL1, CNGA1, NFXL1, LOC112586202; LOC102391616, DNAJB9, THAP5, GPR141, LOC102390377, LOC112586492, LOC112586493; LOC112586881, GPR151, TCERG1, LOC112587225, POU4F3, RBM27, LARS, PLAC8L1; BMP10, LOC102394414, LOC112578097, GKN2, GKN1, ANTXR1, GFPT1, NFU1, AAK1, LOC102411107; LOC102399881, LPIN1, NTSR2, GREB1, E2F6, TRNAE-UUC, LOC112578119, ROCK2, PQLC3, C12H2orf50, KCNF1, LOC112578120, TRNAC-GCA, LOC102412448, PDIA6; LOC112579087; TRNAE-UUC; TRNAE-UUC, LOC112581431, MALT1, ALPK2, LOC102396541,  LOC102390942, LOC102396852, LOC112581366, LOC112581365, NEDD4L, TRNAE-CUC, LOC112581367) | de Araujo Neto et al. (2020) |
|  |  | Nellore (2,273) | BTA3, 5, 6, 21, 26 (U6, CYP4A11, CYP4A22, EFCAB14, DMBX1, LOC513210, FAAH, NSUN4, WIF1, BTC, PARM1, RCHY1, CACUL1, PRDX3, GRK5, RGS10, TIAL1, BAG3) | Do Nascimento et al. (2018) |
|  |  | buffalo (462) | BTA3, 15(SESN3, GPATCH4) | Li et al. (2018) |
|  |  | Brahman cattle (1,796) | TSC22D2, KLF7, ARHGAP29, 7SK, MAP3K5, TLE3, TAF3, WDR5, TMEM68, PPP1R15B, NR2F2, GALR1, SUFU, KCNU1 | Melo *et al.* (2018) |
|  |  | Canchim (392) | BTA4, 27 (NXPH1, EXOC4, ZMAT4) | Buzanskas *et al.* (2017) |
|  |  | Nelore (2,992) | BTA 2, 8, 9, 14, 16, 17 (PAPPA, PREP, FER1L6, TPR, NMNAT1, ACAD10, PCMTD1, CRH, OPKR1, NPBWR1 , NCOA2,STAT1, STAT3, RELA, E2F1,EGR1 et al) | Mota et al. (2017) |
|  |  | Angus, Charolais, Hereford (785) | BTA7,24,25,29 | Akanno et al. (2015) |
|  |  | Buffalo (3,431) | LOC100299005, | Camargo et al. (2015) |
|  |  | Nellore (2,056) | BTA2, 3, 4, 7, 8, 9, 10, 11, 13, 14, 16, 18, 20, 21, 22, 23, 25, 27 (ADAM22, GPR98, FRMD3, LOC784274, SASH1, LOC100847971, LOC785763, SEL1L, GALC, HAAO, KIF16B, CAMK1D, ARMC4, MIR124A2, CYP7B1, LOC782102, SDCCAG8, LOC783434, GTF2H2, OCLN, LOC529061, LOC100847341, FHIT, ELOVL5, LOC514434, MAPK8IP3, RBFOX1, LOC782601, ODZ3, ZNF385D) | Costa et al. (2015) |
|  |  | Hanwoo (96) | BTA1, 2, 7, 10, 14, 16, 20, 26, 29 | KE et al. (2014) |
|  |  | Japanese Black cattle (866) | BTA2 (DNER) | Sasaki et al. (2014) |
|  |  | Holstein (489) | BTA6, 14 | Daetwyler et al. (2008) |
|  | Candidate genes | Sahiwal and Frieswal (250) | OAS1 | Alex *et al.* (2018a) |
|  |  | Sahiwal and Frieswal (250) | OAS1 | Alex *et al.* (2018b) |
|  |  | Holstein (337) | SELP, SELE | Chen et al. (2017) |
|  |  | Nellore (385) | JY1 | Camargo *et al.* (2014) |
|  |  | Slovak Spotted and Pinzgau cows (381) | LEP | Trakovická et al. (2013) |
|  |  | Holstein (518) | STAT5A | Oikonomou et al. (2011) |
|  |  | Holstein (509) | LEP | Clempson *et al.* (2011a) |
| **Non-return** **rate** | GWAS | Holstein (2,527) | BTA4, 11, 19, 23, 27 (LHCGR, FSHR) | Müller et al. (2017) |
|  |  | Brown Swiss cattle (~23,000) | BTA17, 25 (IGLL1, ASCC2, SLC5A4, ENSBTAG00000048030, CABIN1, ENSBTAG00000046900, CDC45L, PYGM, PLCB3) | Frischknecht et al. (2017) |
|  |  | Nordic Red (12,322) | BTA1, 2, 3, 5, 6, 13, 15, 20, 24 (TRPC1, GRK7, EIF4G3, PLA2G2F, RHOC, VAV3, WDR77, GPR125, KCNIP4, SLIT2, SCD5, C4orf22, ANKRD60, GRAMD1B, ZNF521) | Höglund et al. (2015) |
|  |  | Holstein (3,475), Nordic Red (4,998), Jersey (1,225) | BTA4, 13 | Höglund et al. (2014) |
|  |  | Holstein (2,093) | BTA4, 5, 12, 14 (CPT1B, TGFB2, APOH,) | Minozzi et al. (2013) |
|  |  | Holstein (7,937) | BTA7, 8, 11, 19 | Sahana et al. (2013) |
|  |  | Norwegian Red (2,552) | BTA9, 12 | Olsen et al. (2011) |
|  |  | Finish Ayrshire (340) | BTA1, 2, 4, 8, 12, 13, 20, 24 and 27 | Schulman et al. (2011) |
|  |  | Ayrshire (505) | BTA10, 14 | Schulman et al. (2008) |
|  |  | Holstein, Normande and Montbeliarde (4,993) | BTA1, 2, 3 | Ben et al. (2015) |
|  |  | Swedish Red and Holstein (427) | BTA1, 9, 11, 15, 18, 20 | Holmberg and Andersson-Eklund (2006) |
|  | Candidate genes | Holstein-Friesian (309) | LEP | Komisarek (2010) |
|  |  | Simmental (35), Charolais (13) and Limousin (8) | FSHB | Dai et al. (2009) |
|  |  | Holstein-Friesian (453) | PPARGC1A | Komisarek and Dorynek (2009) |
|  |  | Holstein (1,291) | DGAT1 | Kaupe et al. (2007) |
| **Pregnancy** **rate** | GWAS | Nelore (2,273) | BTA6, 26 | Do Nascimento *et al.* (2018) |
|  |  | Holstein (2,107) | Whole genome except BTA20, 23 | Gaddis et al. (2016) |
|  |  | Angus, Charolais, Hereford (785) | BTA9, 20, 21 | Akanno *et al.* (2015) |
|  |  | Holstein (550) | BTA1, 3, 4, 5, 7, 9, 10, 11, 13, 15, 18, 20, 23, 24, 25, (ACAT2, AP3B1, APBB1, ARL6IP1, BSP3, C17H22orf25, C1QB, C7H19orf60, CACNA1D, CAST, CCDC86, CD14, CD40, CFDP2, COQ9, CPSF1, CSNK1E, CSPP1, DEPDC7, DSC2, DYRK3, FUT1, GPLD1, HSD17B12, HSD17B7, LDB3, MARVELD1, MON1B, MRGPRF, MS4A8B, NEU3, NFKBIL1, NLRP9, OCLN, PARM1, PCCB, PMM2, RABEP2, TBC1D24, TDRKH, TSHB, ZP2, DZIP3, FSHR, FYB, GOLGA4) | Cochran et al. (2013) |
|  |  | Holstein (1,654) | BTA7, X (INSR, LOC520057, GRIA3, ATP1B4) | Cole et al. (2011) |
|  |  | Holstein (940) | BTA18 | Muncie et al. (2006) |
|  |  | Holstein (308) | BTA1, 14 | Schnabel et al. (2005) |
|  | Candidate genes | Sahiwal and Frieswal (250) | OAS1 | Alex *et al.* (2018a) |
|  |  | Fleckvieh (786) | LEP, TLR4 | Jecminkova et al. (2018) |
|  |  | Sahiwal and Frieswal (250) | OAS1 | Alex *et al.* (2018b) |
|  |  | Holstein (2,274) | CFDP2, CSPP1, FCER1G, FSHR, GCNT3, IBSP, LHCGR, PMM2, SERPINE2, SREBF1, TBC1D24 | Ortega et al. (2017) |
|  |  | Holstein (2,323) | DZIP3, PCCB, FCER1G, HSD17B7, DNAH11, CSNK1E, TXN2, C7H19orf60, CAST, ACAT2, AP3B1, EPAS1, LHCGR, BCAS1, APBB1, DEPDC7, HSD17B12, MRPL48, PGR, BSP3, COQ9, FUT1, OCLN, BOLA‐DMB, DSC2, ARL6IP1, PMM2, RABEP2, TBC1D24, | Ortega et al. (2016) |
|  |  | Holstein (1,951) | PROP1 | Lan et al. (2013) |
|  |  | Holstein (610) | GH | Mullen et al. (2011) |
|  |  | Holstein (509) | NPY1 | Clempson *et al.* (2011a) |
|  |  | dairy cattle (652) | CAST | Garcia et al. (2006) |

| **Supplement Table3** **Quantitative** **trait** **loci** **(QTLs)** **and** **genes** **associated** **with** **bovine and buffalo calving-related** **traits** | | | | |
| --- | --- | --- | --- | --- |
| **Trait** | **Method** | **Breed** | **Related** **QTLs/genes (located** **chromosomes)** | **Reference** |
| **Calving** **interval** | GWAS | Murrah water buffaloes (765) | BBU 3, 4, 12, 14, 19, 21, 22 (LOC112584001, GNA13, LOC112584002, AMZ2, ARSG, SLC16A6, WIPI1, LOC112583584, PRKAR1A, FAM20A, LOC112583582, LOC112583583, LOC112583585, LOC102395016, LOC102394806, ABCA9, LOC112583586, ABCA6, ABCA10, ABCA5, LOC112584003, MAP2K6, LOC112583587, GLIS3, LOC102414388, LOC112584055, LOC102407374, PPP2R2A, LOC102390311, BNIP3L, PNMA2, DPYSL2, LOC102416107, LOC112584009, ADRA1A, STMN4, TRIM35; CNPY2, PAN2, TRNAE-UUC, LOC112584549, IL23A, STAT2, APOF, NXPH4, LOC102410368, TIMELESS, MIP, LOC112584550, SPRYD4, GLS2, RBMS2, BAZ2A, ATP5F1B, LOC112584805, LOC112584804, PTGES3, LOC102408628, NACA, PRIM1, LOC102407967, TRNAG-CCC, LOC102402888, NDUFA4L2, LOC102407631, LOC102402560, SDR9C7, LOC102390858, LOC102391539, LOC102391871, LOC112577755, LOC102392189, LOC112584350, LOC102392517, LOC102391198, GPR182, ZBTB39, TAC3, MYO1A, NEMP1, LOC102395734, LOC112584551, NAB2, STAT6, LOC102396058, LOC112584798, LRP1, SHMT2, STAC3, LOC112584647, LOC112584356, LOC112584784, E2F7, CSRP2; BMP10, LOC102394414, LOC112578097, GKN2, GKN1, ANTXR1, GFPT1, NFU1, AAK1, LOC102411107; TRNAC-GCA, LOC102404703, FERMT1, LRRN4, CRLS1, MCM8, CHGB,TRMT6, SHLD1, LOC102408362, GPCPD1, PROKR2, LOC102409570, LOC112578916; MTRR, FASTKD3, C19H5orf49, ADCY2; TRNAE-UUC; MAPRE2, LOC102391753, ZNF397, ZSCAN30, LOC102416345, GALNT1, LOC102392092, LOC102416013, INO80C, LOC112581397) | de Araujo Neto *et al.* (2020) |
|  |  | buffalo (462) | BTA3, 6, 10, 12, 23, 28 (PELI2, SOX21, PRDM5, KCNMA1, LMO4) | Li *et al.* (2018) |
|  |  | Holstein (3,729) | BTA13, 21 (FAM181A, SLC24A4, NKX2-1) | Nayeri et al. (2016) |
|  |  | Holstein and Jersey (1,6721) | BTA6 (EPGN) | Raven et al. (2016) |
|  |  | Holstein (7,055) | BTA1, 2, 3, 5, 7, 9, 11, 18, 22, 23, 25, 27, 28, X (ABCC9, EPHA7, LOC101902479, LOC783737, LOC101903002, PRKD2, PPP5C, CACNG6, VSTM1, NR1D2, MYO1B, KCNN3, ABLIM3, OFCC1, GMDS, LOC618542, CHAT) | Aliloo et al. (2015) |
|  |  | Buffalo (4,729) | TPCN1, SCG5 | Camargo *et al.* (2015) |
|  |  | Holstein (7,055) | BTA1, 2, 3, 5, 7, 9, 11, 18, 22, 23, 25, 27, 28, X | Höglund *et al.* (2015) |
|  |  | Gyr Dairy Cattle (2,141) | SMG9, SLC39A11, SOX9 | Machado et al., 2014 |
|  |  | Holstein (2,093) | BTA2, 5, 8, 24, 28 (AMHR2) | Minozzi *et al.* (2013) |
|  |  | Holstein (7,937) | BTA5, 8, 11, 21 | Sahana *et al.* (2013) |
|  |  | Brahman (843) and Tropical Composite (866) | BTA3, 5, 6, 12, 14, 16, 21 | Hawken *et al.* (2012) |
|  |  | Jersey and Limousin (783) | BTA5, 14 (BL37, ILSTS008) | Morris *et al.* (2009) |
|  |  | Holstein (489) | BTA14 | Daetwyler *et al.* (2008) |
|  |  | Holstein-Friesian (770), Charolais (927), Limousin (963) | BTA2, 18 (SIGLEC12, CTU1, ZNF615, PCLO) | Purfield et al. (2015) |
|  | Candidate genes | Holstein (800) | LTF, TLR4 | El-Domany et al. (2019) |
|  |  | Holstein (68) | PON1 | Silveira et al. (2019) |
|  |  | Holstein (410) | GH | Amiri et al. (2018) |
|  |  | Fleckvieh (786) | LEP, TLR4 | Jecminkova *et al.* (2018) |
|  |  | Holstein (337) | SELP, SELE | Chen *et al.* (2017) |
|  |  | Holstein (887) | STAT5A | Hax et al. (2017) |
|  |  | Czech Fleckvieh (419) | DGAT1, BTN1A1 | Rychtářová et al. (2014) |
|  |  | Slovak Spotted (296), Pinzgau (85) | LEP, LEPR | Trakovická *et al.* (2013) |
|  |  | Holstein (509) | LEP | Clempson *et al.* (2011a) |
|  |  | Holstein (431) | GnRH | Derecka et al. (2010) |
|  |  | Holstein (610) | GH | Mullen *et al.* (2011) |
|  |  | Holstein-Friesian (848) | GHR | Waters et al. (2011) |
|  |  | Holstein-Friesian (848) | LTF | O’Halloran et al. (2010) |
| **Days** **open** | GWAS | buffalo (462) | BTA4, 5 (TRHDE, HYAL4, KRR1, MTPN) | Li *et al.* (2018) |
|  |  | Holstein (2,527) | BTA4, 5, 6, 10, 18, 23, 26, 27, 29 | Müller *et al.* (2017) |
|  |  | Holstein (6,734) | BTA1, 4, 26 (PRKG1) | Saowaphak et al. (2017) |
|  |  | Holstein (3,729) | BTA21 (FAM181A) | Nayeri *et al.* (2016) |
|  |  | Japanese Black cattle (459) | BTA2 (PTH2R) | Sasaki et al. (2016) |
|  |  | Holstein (1,371) | NF1 | Sassi et al. (2016) |
|  |  | Buffalo (6,894) | FIG4 | Camargo *et al.* (2015) |
|  |  | Holstein (2,294) | BTA2, 18 (IGFBP2, NDRG4) | Pimentel et al. (2011) |
|  |  | Ayrshire (505) | BTA1, 2, 5, 12, 20, 25, 29 | Schulman *et al.* (2008) |
|  | Candidate genes | Holstein (800) | LTF, TLR4 | El-Domany *et al.* (2019) |
|  |  | Fleckvieh (786) | LEP | Jecminkova *et al.* (2018) |
|  |  | Holstein (410) | GH | Amiri *et al.* (2018) |
|  |  | Holstein (659) | GHR, PAPPA2, PRLR, SOCS4 | Leyva-Corona et al. (2018) |
|  |  | Holstein (337) | SELP, SELE | Chen *et al.* (2017) |
|  |  | Holstein (2,274) | BCAS1, BDH2, BSP3, CAST, CD14, CD2, CSPP1, FCER1G, FUT1, FYB, HSD17B7, IBSP, NLRP9, OCLN, PCCB, PMM2, RABEP2, TBC1D24, TSHB | Ortega *et al.* (2017) |
|  |  | Holstein (123) | CAST, DGAT1 | Hill et al. (2016) |
|  |  | Czech Fleckvieh (419) | DGAT1, BTN1A1 | Rychtářová *et al.* (2014) |
|  |  | Holstein (408) | SCD1 | Asadollahpour Nanaei et al. (2014) |
|  |  | Holstein (204) | TNF | Shirasuna et al. (2010) |
|  |  | Holstein (509) | LEP, NPY1 | Clempson *et al.* (2011a) |
| **Calving** **difficulty** | GWAS | Holstein (8,780), Brown Swiss (505), Jersey (1,818) | BTA4, 5, 6, 9, 15, 17, 18, 25, 29 | Tiezzi et al. (2018) |
|  |  | Nelore (2,273) | BTA6, 26 | Do Nascimento *et al.* (2018) |
|  |  | Brown Swiss cattle (~23,000) | BTA5, 19, 21, 22, 25, 29 (CRAMP1, LPTX4, TELO2, TUBD1) | Frischknecht *et al.* (2017) |
|  |  | Holstein (2,527) | BTA7, 18 (RLN3) | Müller *et al.* (2017) |
|  |  | Angus, Charolais, Hereford (785) | BTA5, 14, 16 | Akanno *et al.* (2015) |
|  |  | Nordic Red (4,631) | BTA6 (LCORL, NCAPG) | Sahana et al. (2015) |
|  |  | Holstein-Friesian (770), Charolais (927), Limousin (963) | BTA2, 18 (TMEM194B, INPP1, MFDS6, C2H2orf88, HIBCH, MSTN, PMS1, PCLO, ORMDL1, OSGEPL1, SIRPA, PDYN, ANKAR and ASNSD1) | Purfield *et al.* (2015) |
|  |  | Piedmontese (323) | BTA6 (LAP3, NCAPG, LCORL) | Bongiorni et al. (2012) |
|  |  | German Fleckvieh (1,829) | BTA14, 21 (RPS20) | Pausch et al. (2011) |
|  |  | Holstein (1,654) | BTA18 (PGLYRP1, IGFL1, LOC787057) | Cole *et al.* (2011) |
|  |  | Angus (1,769) | BTA1, 5, 6, 8, 10, 14, 16, 17, 19, 23, 25 (TG) | McClure et al. (2010) |
|  |  | Norwegian Red (2,552) | BTA4, 5, 6, 9, 12, 20, 22, 28 (SPP1, MEPE, IBSP, LAP3, MED28, NCAPG, WHITE, ABCG2) | Olsen et al. (2010) |
|  |  | Norwegian Red (731) | BTA6 (OPN, MEPE, IBSP, LAP3, EG1, NCAPG) | Olsen et al. (2008) |
|  |  | Ayrshire (505) | BTA4, 6, 11, 15, 18, 23 | Schulman *et al.* (2008) |
|  | Candidate genes | Sahiwal and Frieswal (250) | OAS1 | Alex *et al.* (2018a) |
|  |  | Sahiwal and Frieswal (250) | OAS1 | Alex *et al.* (2018b) |
|  |  | Holstein (848) | GNAS | Sikora et al. (2011) |
|  |  | Holstein (1,664) | PAPPA2 | Wickramasinghe et al. (2011) |
|  |  | Holstein (368) | PGF | Seidenspinner et al. (2011) |
|  |  | Holstein-Friesian (385) | LEP | Brickell et al. (2010) |
|  |  | Holstein (848) | LEP | Giblin et al. (2010) |
|  |  | Holstein (848) | PEG3, ZIM2, TSPAN32 | Magee et al. (2010) |
|  |  | Holstein (1,291) | CYP11B1 | Kaupe *et al.* (2007) |
|  |  | Piedmontese (302) | MSTN | Casas et al. (1999) |
| **Length** **of** **productive** **life** | GWAS | 1/2 Red Angus, 1/4 Charolais, 1/4 Tarentaise (547) | BTA1, 2, 3, 8, 9, 11, 12, 14, 15, 19, 20, 21, 25, 27, 29 (GBE1, GTDC1, ZEB2, CTNNA2, REG3A, REG3G, BTBD17, CD300A, CD300LB, CDR2L, FADS6, FDXR, GPRC5C, GRIN2C, HID1) | Hamidi Hay and Roberts (2017) |
|  |  | Holstein (5,314), Norwegian Red (4,200), Jersey (1,061) | BTA5, 6, 9, 10, 18, 21, 23, 25 (NPFFR2, GC, ZNF717, ZNF613) | Zhang et al. (2016) |
|  |  | Fleckvieh (4,887) | BTA6, 13, 14, 19, 21 (DERL1, SNTG1, LOC614437, E2F1, RALY, SYT10, NTRK2, FOXO3, LOC519798, LOC100140952, APOE, ADAMTS3) | Mészáros et al. (2014) |
|  |  | Holstein (550) | ACAT2, AP3B1, ARL6IP1, ASL, AVP, CCDC86, CD40, CFDP2, COQ9, CSPP1, DEPDC7, DSC2, FSHR, FUT1, GPLD1, HSD17B12, HSD17B6, HSD17B7, HSPA1A, IBSP, LDB3, LHCGR, MARVELD1, MON1B, MS4A8B, NEU3, OCLN, PARM1, PCCB, PMM2, RABEP2, SYTL2, TBC1D24, TDRKH, WBP1, ZP2 | Cochran *et al.* (2013) |
|  |  | Holstein (1,654) | BTA7, X (INSR, LOC520057) | Cole *et al.* (2011) |
|  |  | Holstein (940) | BTA18 | Muncie *et al.* (2006) |
|  | Candidate genes | Holstein (337) | SELP | Chen *et al.* (2017) |
|  |  | Holstein (192) | CSN3 | Lukač et al. (2015) |
|  |  | Holstein (1,951) | PROP1 | Lan *et al.* (2013) |
|  |  | Holstein (509) | TFAM, UCP2 | Clempson et al. (2011b) |
|  |  | Holstein (1,664) | PAPPA2 | Wickramasinghe *et al.* (2011) |
|  |  | Holstein (848) | LEP | Giblin *et al.* (2010) |
|  |  | Holstein (2,773) | FGF2 | Wang et al. (2008) |
|  |  | Holstein (842) | POU1F1 | Huang et al. (2008b) |
|  |  | Holstein (1,291) | CYP11B1, DGAT1 | Kaupe *et al.* (2007) |
|  |  | Holstein (1,390) | UTMP | Khatib et al. (2007) |
|  |  | dairy cattle (652) | CAST | Garcia *et al.* (2006) |
|  |  | Holstein (1,258) | PI | Khatib et al. (2005) |


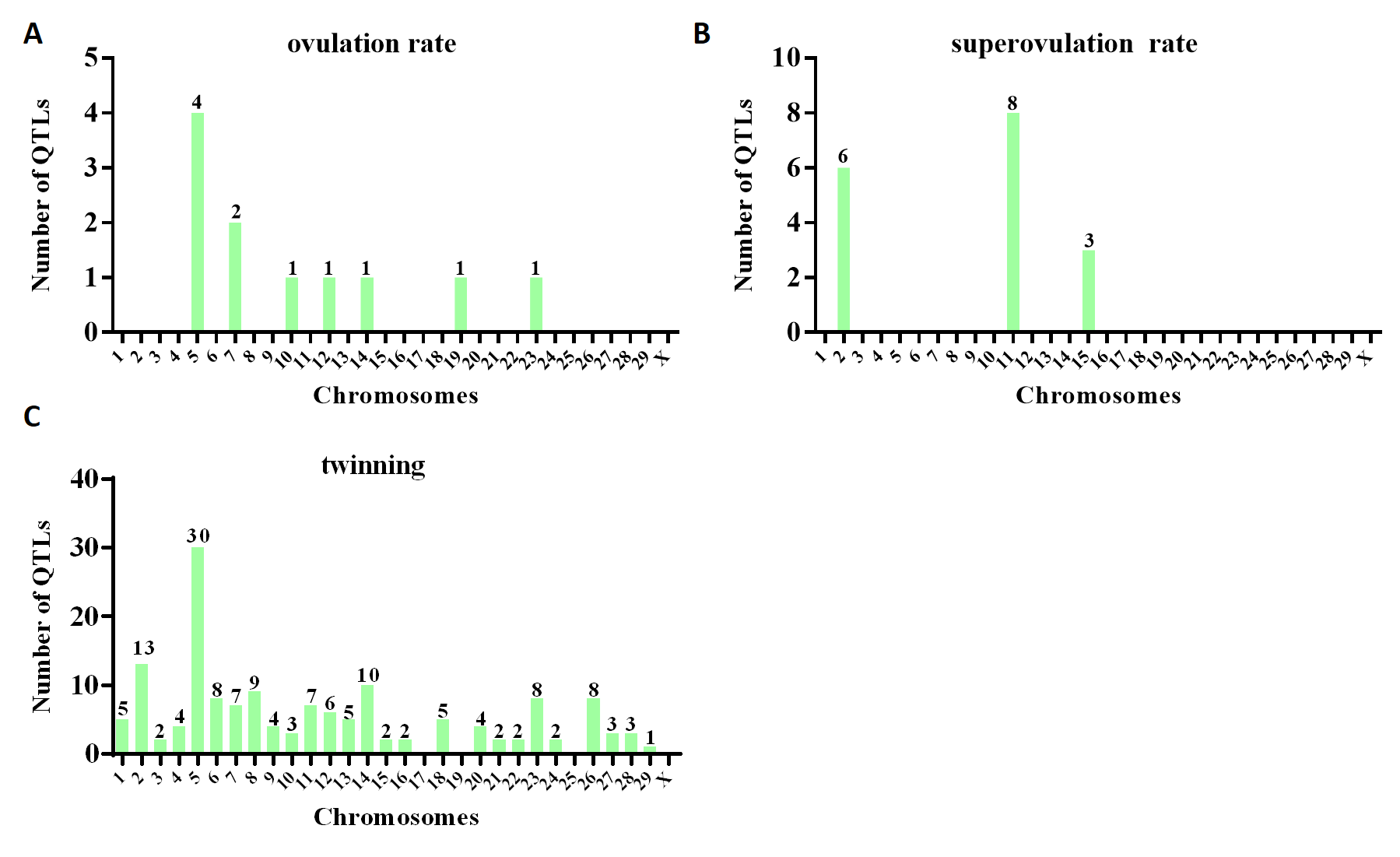


**Supplemental Figure 1. Distribution of reported quantitative trait loci (QTL) for bovine ovulation-related traits**

**
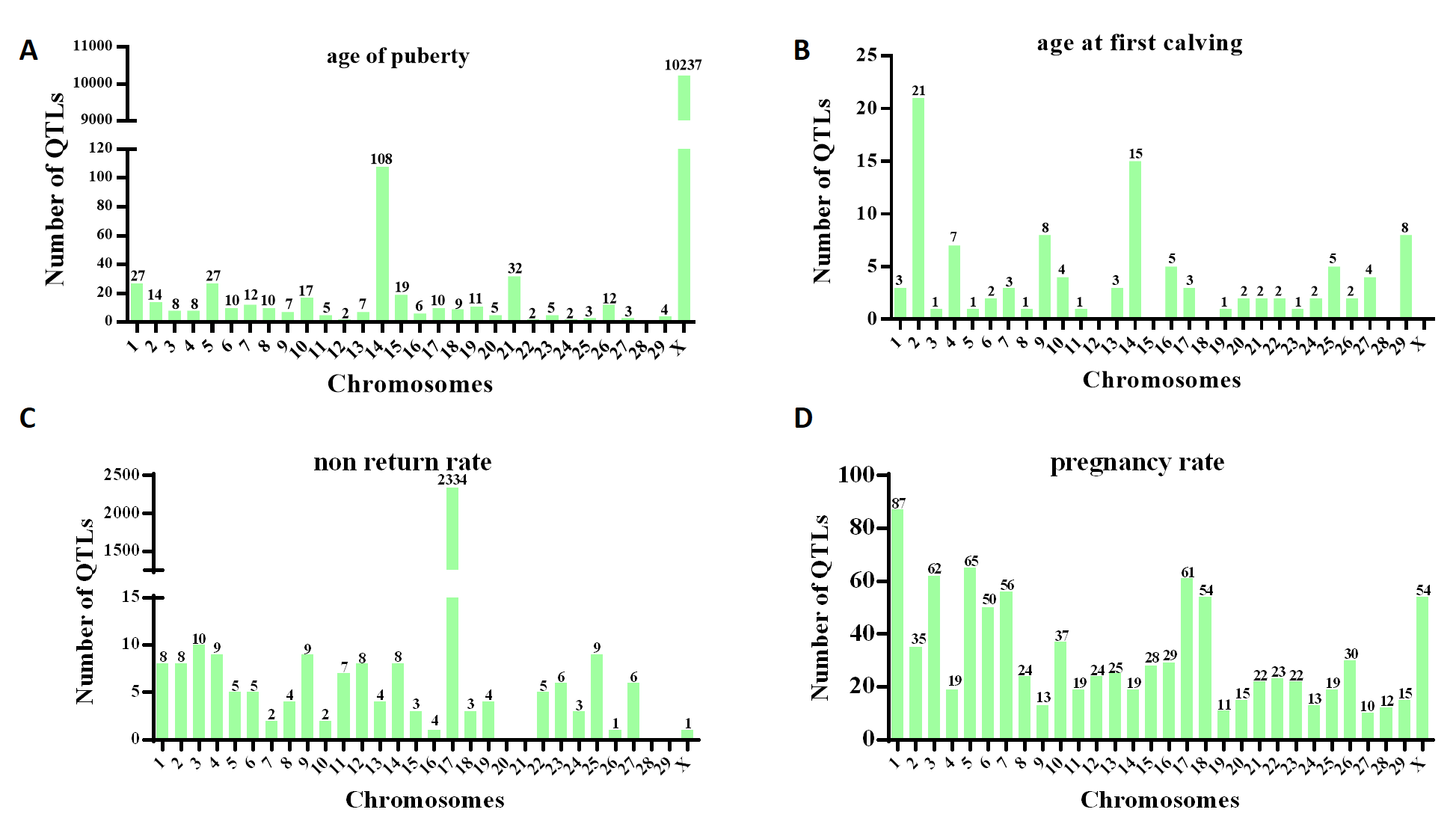
**

**Supplemental Figure 2. Distribution of reported quantitative trait loci (QTL) for cattle mating-related traits**

**
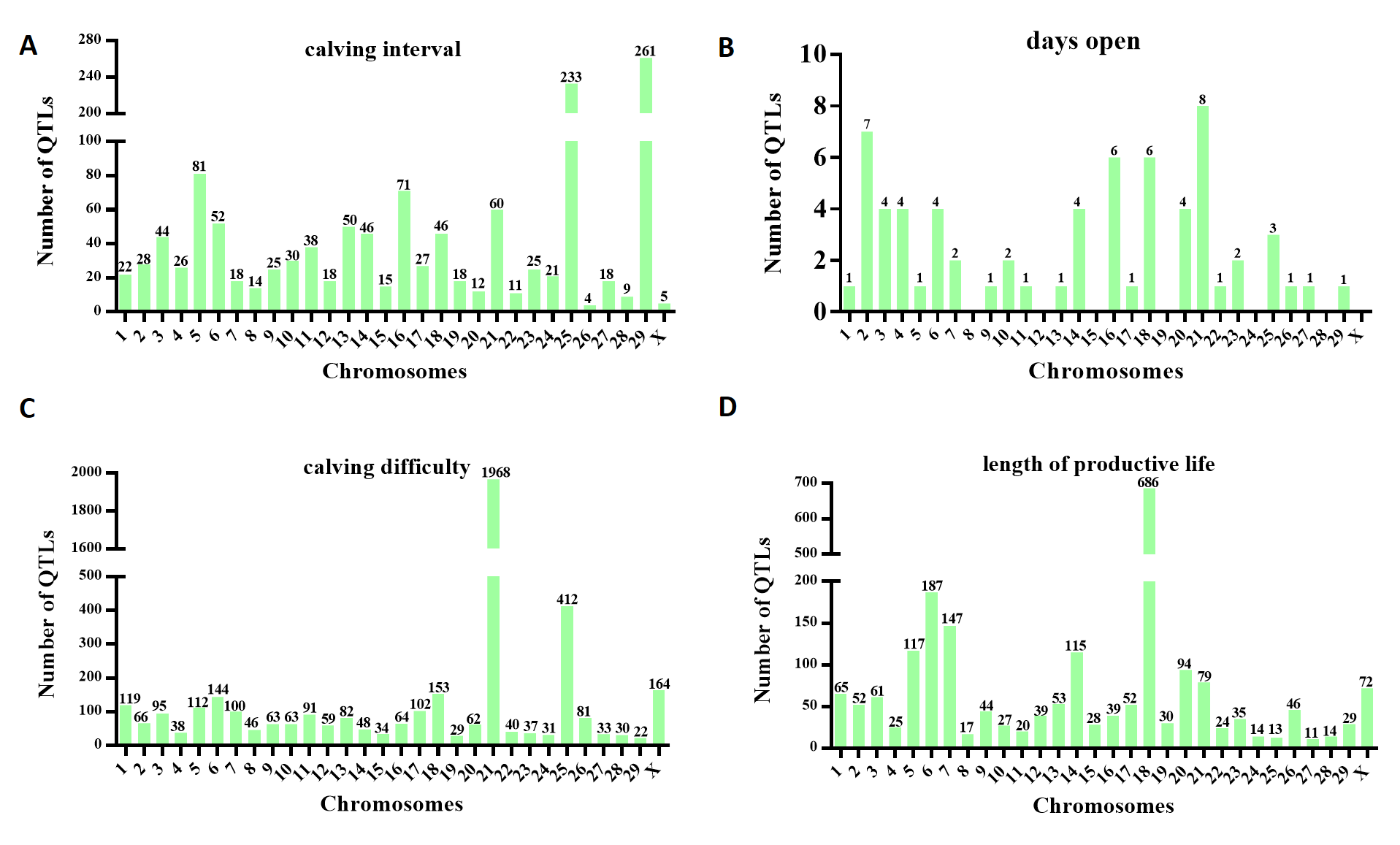
**

**Supplemental Figure 3. Distribution of reported quantitative trait loci (QTL) for cattle calving-related traits**

**Reference:**

1. Akanno E.C., Plastow G., Fitzsimmons C., Miller S.P., Baron V., Ominski K. & Basarab J.A. (2015) Genome-wide association for heifer reproduction and calf performance traits in beef cattle. Genome 58, 549-57.

2. Alex R., Ramesha K., Singh U., Kumar S., Alyethodi R.R., Deb R., Sharma S., Sengar G.S., Kumar A. & Prakash B. (2018a) Promoter variants of OAS1 gene are associated with reproductive performance and incidence of normal calving in cattle. Theriogenology 108, 255-61.

3. Alex R., Ramesha K.P., Singh U., Kumar S. & Prakash B. (2018b) Association analysis of novel polymorphisms in 2′, 5′-oligoadenylate synthetase gene with reproductive traits in indigenous and cross-bred cattle of Indian Origin. Reproduction in Domestic Animals 53.

4. Aliloo H., Pryce J.E., González-Recio O., Cocks B.G. & Hayes B.J. (2015) Validation of markers with non-additive effects on milk yield and fertility in Holstein and Jersey cows. Bmc Genetics 16, 89.

5. Allan M., Kuehn L., Cushman R., Snelling W., Echternkamp S. & Thallman R. (2014) Confirmation of quantitative trait loci using a low-density single nucleotide polymorphism map for twinning and ovulation rate on bovine chromosome 5. Journal of animal science 87, 46-56.

6. Allan M., Thallman R., Cushman R., Echternkamp S., White S., Kuehn L., Casas E. & Smith T. (2007) Association of a single nucleotide polymorphism in SPP1 with growth traits and twinning in a cattle population selected for twinning rate. Journal of animal science 85, 341-7.

7. Amiri S., Jemmali B., Ferchichi M.A., Jeljeli H., Boulbaba R. & Ben Gara A. (2018) Assessment of growth hormone gene polymorphism effects on reproductive traits in Holstein dairy cattle in Tunisia. Archives Animal Breeding 61, 481-9.

8. Arias J. & Kirkpatrick B. (2004) Mapping of bovine ovulation rate QTL; an analytical approach for three generation pedigrees. Animal Genetics 35, 7-13.

9. Arslan K., Akyüz B., Akçay A., İlgar E.G., Macun H.C. & Çınar M.U. (2017) Association of number of artificial inseminations per pregnancy in holstein dairy cows with polymorphism in luteinizing hormone receptor and follicle stimulating hormone receptor genes. Slovenian Veterinary Research 54, 91-8.

10. Asadollahpour Nanaei H., Ansari Mahyari S. & Edriss M.A. (2014) Effect of LEPR, ABCG 2 and SCD 1 Gene Polymorphisms on Reproductive Traits in the Iranian Holstein Cattle. Reproduction in Domestic Animals 49, 769-74.

11. Ben J.S., Fritz S., Guillaume F., Druet T., Denis C., Eggen A. & Gautier M. (2015) Detection of quantitative trait loci affecting non-return rate in French dairy cattle. Journal of Animal Breeding & Genetics 125, 280-8.

12. Bierman C.D., Kim E., ., Weigel K., ., Berger P.J. & Kirkpatrick B.W. (2010a) Fine-mapping quantitative trait loci for twinning rate on Bos taurus chromosome 14 in North American Holsteins. Journal of Animal Science 88, 2556-64.

13. Bierman C.D., Kim E., Shi X.W., Weigel K., Jeffrey B.P. & Kirkpatrick B.W. (2010b) Validation of whole genome linkage-linkage disequilibrium and association results, and identification of markers to predict genetic merit for twinning. Animal Genetics 41, 406-16.

14. Blattman A., Kirkpatrick B. & Gregory K. (1996) A search for quantitative trait loci for ovulation rate in cattle. Animal Genetics 27, 157-62.

15. Bongiorni S., Mancini G., Chillemi G., Pariset L. & Valentini A. (2012) Identification of a short region on chromosome 6 affecting direct calving ease in Piedmontese cattle breed. Plos One 7, e50137.

16. Brickell J., Pollott G., Clempson A., Otter N. & Wathes D. (2010) Polymorphisms in the bovine leptin gene associated with perinatal mortality in Holstein-Friesian heifers. Journal of Dairy Science 93, 340-7.

17. Buzanskas M.E., do Amaral Grossi D., Ventura R.V., Schenkel F.S., Chud T.C.S., Stafuzza N.B., Rola L.D., Meirelles S.L.C., Mokry F.B. & de Alvarenga Mudadu M. (2017) Candidate genes for male and female reproductive traits in Canchim beef cattle. Journal of animal science and biotechnology 8, 67.

18. Camargo G.D., Aspilcueta-Borquis R.R., Fortes M., Porto-Neto R., Cardoso D.F., Santos D., Lehnert S.A., Reverter A., Moore S.S. & Tonhati H. (2015) Prospecting major genes in dairy buffaloes. Bmc Genomics 16, 1-14.

19. Camargo G.M.F., De, Raphael Bermal C., Lucia Galv?O D.A., Fernando B. & Humberto T. (2014) Association between JY-1 gene polymorphisms and reproductive traits in beef cattle. Gene 533, 477-80.

20. Casas E., Keele J.W., Fahrenkrug S.C., Smith T.P., Cundiff L.V. & Stone R.T. (1999) Quantitative analysis of birth, weaning, and yearling weights and calving difficulty in Piedmontese crossbreds segregating an inactive myostatin allele. Journal of Animal Science 77, 1686-92.

21. Chen X., Zhang S., Cheng Z., Cooke J.S., Werling D., Wathes D.C. & Pollott G.E. (2017) Polymorphisms in the selectin gene cluster are associated with fertility and survival time in a population of Holstein Friesian cows. Plos One 12, e0175555.

22. Clempson A., Pollott G., Brickell J., Bourne N., Munce N. & Wathes D. (2011a) Evidence that leptin genotype is associated with fertility, growth, and milk production in Holstein cows. Journal of Dairy Science 94, 3618-28.

23. Clempson A., Pollott G., Brickell J., Bourne N., Munce N. & Wathes D. (2011b) Polymorphisms in the autosomal genes for mitochondrial function TFAM and UCP2 are associated with performance and longevity in dairy cows. animal 5, 1335-43.

24. Cobanoglu O., Berger P.J. & Kirkpatrick B.W. (2005) Genome screen for twinning rate QTL in four North American Holstein families. Animal Genetics 36, 303-8.

25. Cochran S.D., Cole J.B., Null D.J. & Hansen P.J. (2013) Discovery of single nucleotide polymorphisms in candidate genes associated with fertility and production traits in Holstein cattle. BMC genetics 14, 49.

26. Cole J.B., Wiggans G.R., Ma L., Sonstegard T.S., Lawlor T.J., Crooker B.A., Tassell C.P.V., Yang J., Wang S. & Matukumalli L.K. (2011) Genome-wide association analysis of thirty one production, health, reproduction and body conformation traits in contemporary U.S. Holstein cows. Bmc Genomics 12, 408.

27. Collis E., Fortes M.R., Zhang Y., Tier B., Schutt K., Barendse W. & Hawken R. (2012) Genetic variants affecting meat and milk production traits appear to have effects on reproduction traits in cattle. Animal Genetics 43, 442-6.

28. Cory A.T., Price C.A., Lefebvre R. & Palin M.F. (2013) Identification of single nucleotide polymorphisms in the bovine follicle-stimulating hormone receptor and effects of genotypes on superovulatory response traits. Animal Genetics 44, 197-201.

29. Costa R.B., Camargo G.M., Diaz I.D., Irano N., Dias M.M., Carvalheiro R., Boligon A.A., Baldi F., Oliveira H.N. & Tonhati H. (2015) Genome-wide association study of reproductive traits in Nellore heifers using Bayesian inference. Genetics Selection Evolution,47,1(2015-08-19) 47, 67.

30. Cruickshank J., Dentine M.R., Berger P.J. & Kirkpatrick B.W. (2004) Evidence for quantitative trait loci affecting twinning rate in North American Holstein cattle. Animal Genetics 35, 206-12.

31. Daetwyler H.D., Schenkel F.S., Sargolzaei M. & Robinson J.A.B. (2008) A Genome Scan to Detect Quantitative Trait Loci for Economically Important Traits in Holstein Cattle Using Two Methods and a Dense Single Nucleotide Polymorphism Map. Journal of Dairy Science 91, 3225-36.

32. Dai L., Zhao Z., Zhao R., Xiao S., Jiang H., Yue X., Li X., Gao Y., Liu J. & Zhang J. (2009) Effects of novel single nucleotide polymorphisms of the FSH beta-subunit gene on semen quality and fertility in bulls. Animal Reproduction Science 114, 14-22.

33. de Araujo Neto F.R., Takada L., Dos Santos D.J.A., Aspilcueta‐Borquis R.R., Cardoso D.F., do Nascimento A.V., Leão K.M., de Oliveira H.N. & Tonhati H. (2020) Identification of genomic regions related to age at first calving and first calving interval in water buffalo using single‐step GBLUP. Reproduction in Domestic Animals 55, 1565-72.

34. Derecka K., Ahmad S., Hodgman T.C., Hastings N., Royal M.D., Woolliams J.A. & Flint A.P.F. (2010) Sequence variants in the bovine gonadotrophin releasing hormone receptor gene and their associations with fertility. Animal Genetics 41, 329-31.

35. Do Nascimento A.V., da Silva Romero Â.R., Utsunomiya Y.T., Utsunomiya A.T.H., Cardoso D.F., Neves H.H.R., Carvalheiro R., Garcia J.F. & Grisolia A.B. (2018) Genome-wide association study using haplotype alleles for the evaluation of reproductive traits in Nelore cattle. Plos One 13, e0201876.

36. El-Domany W.B., Radwan H.A., Ateya A.I., Ramadan H.H., Marghani B.H. & Nasr S.M. (2019) Genetic Polymorphisms in LTF/EcoRI and TLR4/AluI loci as candidates for milk and reproductive performance assessment in Holstein cattle. Reproduction in Domestic Animals 54, 678-86.

37. Fernandez M.E., Loaiza Echeverri A., Henry M., Drummond M., Andrade de Oliveira D., Demyda Peyrás S., Cunha Cardoso D., Giovambattista G. & Liron J.P. (2017) Bovine thyroglobulin gene polymorphisms and their association with sexual precocity in Guzerat bulls. Reproduction in Domestic Animals 52, 911-3.

38. Fortes M., Lehnert S., Bolormaa S., Reich C., Fordyce G., Corbet N., Whan V., Hawken R. & Reverter A. (2012) Finding genes for economically important traits: Brahman cattle puberty. Animal Production Science 52, 143-50.

39. Fortes M.R., Reverter A., Kelly M., Mcculloch R. & Lehnert S.A. (2013) Genome-wide association study for inhibin, luteinizing hormone, insulin-like growth factor 1, testicular size and semen traits in bovine species. Andrology 1, 644-50.

40. Frischknecht M., Bapst B., Seefried F.R., Signer-Hasler H., Garrick D., Stricker C., Fries R., Russ I., Sölkner J. & Bieber A. (2017) Genome-wide association studies of fertility and calving traits in Brown Swiss cattle using imputed whole-genome sequences. Bmc Genomics 18, 910.

41. Gaddis K.L.P., Null D.J. & Cole J.B. (2016) Explorations in genome-wide association studies and network analyses with dairy cattle fertility traits. Journal of Dairy Science 99, 6420-35.

42. Gaddis K.P., Dikmen S., Null D., Cole J. & Hansen P. (2017) Evaluation of genetic components in traits related to superovulation, in vitro fertilization, and embryo transfer in Holstein cattle. Journal of dairy science 100, 2877-91.

43. Garcia M., Michal J., Gaskins C., Reeves J., Ott T., Liu Y. & Jiang Z. (2006) Significant association of the calpastatin gene with fertility and longevity in dairy cattle. Animal Genetics 37, 304-5.

44. Giblin L., Butler S.T., Kearney B.M., Waters S.M., Callanan M.J. & Berry D.P. (2010) Association of bovine leptin polymorphisms with energy output and energy storage traits in progeny tested Holstein-Friesian dairy cattle sires. Bmc Genetics 11, 73.

45. Gonda M.G., Arias J.A., Shook G.E. & Kirkpatrick B.W. (2004) Identification of an ovulation rate QTL in cattle on BTA14 using selective DNA pooling and interval mapping. Animal Genetics 35, 298-304.

46. Hamidi Hay E. & Roberts A. (2017) Genomic prediction and genome-wide association analysis of female longevity in a composite beef cattle breed. Journal of Animal Science 95, 1467-71.

47. Hawken R.J., Zhang Y.D., Fortes M.R.S., Collis E., Barris W.C., Corbet N.J., Williams P.J., Fordyce G., Holroyd R.G. & Walkley J.R.W. (2012) Genome-wide association studies of female reproduction in tropically adapted beef cattle. Journal of Animal Science 90, 1398-410.

48. Hax L.T., Schneider A., Jacometo C.B., Mattei P., Silva T.C.d., Farina G. & Corrêa M.N. (2017) Association between polymorphisms in somatotropic axis genes and fertility of Holstein dairy cows. Theriogenology 88, 67-72.

49. Hill R., Canal A., Bondioli K., Morell R. & Garcia M.D. (2016) Molecular markers located on the DGAT1, CAST, and LEPR genes and their associations with milk production and fertility traits in Holstein cattle. Genetics and Molecular Research 15.

50. Höglund J.K., Buitenhuis B., Guldbrandtsen B., Lund M.S. & Sahana G. (2015) Genome-wide association study for female fertility in Nordic Red cattle. Bmc Genetics 16, 110.

51. Höglund J.K., Sahana G., Brøndum R.F., Guldbrandtsen B., Buitenhuis B. & Lund M.S. (2014) Fine mapping QTL for female fertility on BTA04 and BTA13 in dairy cattle using HD SNP and sequence data. Bmc Genomics 15, 790.

52. Holmberg M., . & Andersson-Eklund L., . (2006) Quantitative trait loci affecting fertility and calving traits in Swedish dairy cattle. Journal of Dairy Science 89, 3664-71.

53. Huang M., Shang-Zhong X.U., Zan L.S., Zhang L.P. & Gao X. (2008a) Genetic variation in RXRG gene and its relationship with twinning trait in cattle. Hereditas 30, 190-4.

54. Huang W., Maltecca C. & Khatib H. (2008b) A proline‐to‐histidine mutation in POU1F1 is associated with production traits in dairy cattle. Animal Genetics 39, 554-7.

55. Jaton C., Schenkel F., Sargolzaei M., Cánova A., Malchiodi F., Price C., Baes C. & Miglior F. (2018) Genome-wide association study and in silico functional analysis of the number of embryos produced by Holstein donors. Journal of Dairy Science 101, 7248-57.

56. Jecminkova K., Müller U., Kyselova J., Sztankoova Z., Zavadilova L., Stipkova M. & Majzlik I. (2018) Association of leptin, toll-like receptor 4, and chemokine receptor of interleukin 8 CXC motif single nucleotide polymorphisms with fertility traits in Czech Fleckvieh cattle. Asian-australasian journal of animal sciences 31, 1721.

57. Kappes S., Bennett G., Keele J., Echternkamp S., Gregory K. & Thallman R. (2000) Initial results of genomic scans for ovulation rate in a cattle population selected for increased twinning rate. Journal of Animal Science 78, 3053-9.

58. Kaupe B., Brandt H., Prinzenberg E. & Erhardt G. (2007) Joint analysis of the influence of CYP11B1 and DGAT1 genetic variation on milk production, somatic cell score, conformation, reproduction, and productive lifespan in German Holstein cattle. Journal of animal science 85, 11-21.

59. KE H., Iqbal A. & JJ K. (2014) A Genome Wide Association Study on Age at First Calving Using High Density Single Nucleotide Polymorphism Chips in Hanwoo (Bos taurus coreanae). 27, 1406–10.

60. Khatib H., Heifetz E. & Dekkers J. (2005) Association of the protease inhibitor gene with production traits in Holstein dairy cattle. Journal of Dairy Science 88, 1208-13.

61. Khatib H., Schutzkus V., Chang Y. & Rosa G. (2007) Pattern of expression of the uterine milk protein gene and its association with productive life in dairy cattle. Journal of Dairy Science 90, 2427-33.

62. Kim E.-S., Shi X., Cobanoglu O., Weigel K., Berger P. & Kirkpatrick B. (2009a) Refined mapping of twinning-rate quantitative trait loci on bovine chromosome 5 and analysis of insulin-like growth factor-1 as a positional candidate gene. Journal of Animal Science 87, 835-43.

63. Kim E.S., Berger P. & Kirkpatrick B. (2009b) Genome‐wide scan for bovine twinning rate QTL using linkage disequilibrium. Animal Genetics 40, 300-7.

64. Kirkpatrick B.W., Byla B.M. & Gregory K.E. (2000) Mapping quantitative trait loci for bovine ovulation rate. Mammalian Genome 11, 136-9.

65. Kirkpatrick B.W. & Morris C.A. (2015) Mapping of a major gene for bovine ovulation rate. Plos One 10, 13.

66. Komisarek J. (2010) Impact of LEP and LEPR gene polymorphisms on functional traits in Polish Holstein-Friesian cattle. Animal Science Papers & Reports 28, 133-41.

67. Komisarek J. & Dorynek Z. (2002) Genetic aspects of twinning in cattle. Journal of applied genetics 43, 55-68.

68. Komisarek J. & Dorynek Z. (2009) Effect ofABCG2, PPARGC1A, OLR1 andSCD1 gene polymorphism on estimated breeding values for functional and production traits in Polish Holstein-Friesian bulls. Journal of applied genetics 50, 125-32.

69. Lan X., Peñagaricano F., DeJung L., Weigel K. & Khatib H. (2013) A missense mutation in the PROP1 (prophet of Pit 1) gene affects male fertility and milk production traits in the US Holstein population. Journal of Dairy Science 96, 1255-7.

70. Leyva-Corona J.C., Reyna-Granados J.R., Ricardo Z.A., Sanchez-Castro M.A., Thomas M.G., Mark E.R., Speidel S.E., Medrano J.F., Gonzalo R. & Pablo L.N. (2018) Polymorphisms within the prolactin and growth hormone/insulin-like growth factor-1 functional pathways associated with fertility traits in Holstein cows raised in a hot-humid climate. Tropical animal health and production 50, 1913-20.

71. Li J., Liu J., Campanile G., Plastow G., Zhang C., Wang Z., Cassandro M., Gasparrini B., Salzano A. & Hua G. (2018) Novel insights into the genetic basis of buffalo reproductive performance. BMC genomics 19, 814.

72. Lien S., Karlsen A., Klemetsdal G., Våge D.I., Olsaker I., Klungland H., Aasland M., Heringstad B., Ruane J. & Gomezraya L. (2000) A primary screen of the bovine genome for quantitative trait loci affecting twinning rate. Mammalian Genome 11, 877-82.

73. Lirón J.P., Prando A.J., Fernández M.E., Ripoli M.V., Rogberg-Muñoz A., Goszczynski D.E., Posik D.M., Peral-García P., Baldo A. & Giovambattista G. (2012) Association between GNRHR, LHR and IGF1 polymorphisms and timing of puberty in male Angus cattle. Bmc Genetics 13, 26.

74. Liu A., Wang Y., Sahana G., Zhang Q., Liu L., Lund M.S. & Su G. (2017) Genome-wide Association Studies for Female Fertility Traits in Chinese and Nordic Holsteins. Scientific Reports 7, 8487.

75. Lukač D., Jovanovac S., Nemes Z., Vidović V., Popović-Vranješ A., Raguž N. & Lopičić-Vasić T. (2015) Association of polymorphism κ-casein gene with longevity and lifetime production of Holstein-Friesian cows in Vojvodina. Mljekarstvo 65, 232-7.

76. Magee D.A., Sikora K.M., Berkowicz E.W., Berry D.P., Howard D.J., Mullen M.P., Evans R.D., Spillane C. & MacHugh D.E. (2010) DNA sequence polymorphisms in a panel of eight candidate bovine imprinted genes and their association with performance traits in Irish Holstein-Friesian cattle. Bmc Genetics 11, 93.

77. Marchitelli C. & Nardone A. (2015) Mutations and sequence variants in GDF9, BMP15, and BMPR1B genes in Maremmana cattle breed with single and twin births. Rendiconti Lincei 26, 553-60.

78. Mayumi S., Shinji S., Toshio W., Shota N., Atsushi I., Maya Y., Keiko M., Michisuke Y., Kenji S. & Yoshito A. (2010) Ionotropic glutamate receptor AMPA 1 is associated with ovulation rate. Plos One 5, e13817.

79. McClure M., Morsci N., Schnabel R., Kim J., Yao P., Rolf M., McKay S., Gregg S., Chapple R. & Northcutt S. (2010) A genome scan for quantitative trait loci influencing carcass, post‐natal growth and reproductive traits in commercial Angus cattle. Animal genetics 41, 597-607.

80. Melo T.P., Fortes M.R., Bresolin T., Mota L.F., Albuquerque L.G. & Carvalheiro R. (2018) Multitrait meta-analysis identified genomic regions associated with sexual precocity in tropical beef cattle. Journal of Animal Science 96, 4087-99.

81. Mészáros G., Eaglen S., Waldmann P. & Sölkner J. (2014) A genome wide association study for longevity in cattle. Open Journal of Genetics 4, 46.

82. Meuwissen T.H.E., Astrid K., SigbjøRn L., Ingrid O. & Goddard M.E. (2002) Fine mapping of a quantitative trait locus for twinning rate using combined linkage and linkage disequilibrium mapping. Genetics 161, 373.

83. Minozzi G., Nicolazzi E.L., Stella A., Biffani S., Negrini R., Lazzari B., Ajmone-Marsan P. & Williams J.L. (2013) Genome wide analysis of fertility and production traits in Italian Holstein cattle. Plos One 8, e80219.

84. Morris C.A., Pitchford W.S., Cullen N.G., Esmailizadeh A.K., Hickey S.M., Hyndman D., Dodds K.G., Afolayan R.A., Crawford A.M. & Bottema C.D.K. (2009) Quantitative trait loci for live animal and carcass composition traits in Jersey and Limousin back-cross cattle finished on pasture or feedlot. Animal Genetics 40, 648-54.

85. Mota R., Guimarães S., Fortes M., Hayes B., Silva F., Verardo L., Kelly M., de Campos C., Guimarães J. & Wenceslau R. (2017) Genome‐wide association study and annotating candidate gene networks affecting age at first calving in Nellore cattle. Journal of animal breeding and genetics 134, 484-92.

86. Mullen M.P., Lynch C.O., Waters S.M., Howard D.J., O'Boyle P., Kenny D.A., Buckley F., Horan B. & Diskin M.G. (2011) Single nucleotide polymorphisms in the growth hormone and insulin-like growth factor-1 genes are associated with milk production, body condition score and fertility traits in dairy cows. Genetics & Molecular Research 1, 1819-30.

87. Müller M.P., Rothammer S., Seichter D., Russ I., Hinrichs D., Tetens J., Thaller G. & Medugorac I. (2017) Genome-wide mapping of 10 calving and fertility traits in Holstein dairy cattle with special regard to chromosome 18. Journal of Dairy Science 100, 1987-2006.

88. Muncie S., Cassady J. & Ashwell M. (2006) Refinement of quantitative trait loci on bovine chromosome 18 affecting health and reproduction in US Holsteins. Animal Genetics 37, 273-5.

89. Nascimento A.V., Matos M.C., Seno L.O., Romero A.R., Garcia J.F. & Grisolia A.B. (2016) Genome wide association study on early puberty in Bos indicus. Genetics & Molecular Research 15, 1-6.

90. Nayeri S., Sargolzaei M., Abo-Ismail M.K., May N., Miller S.P., Schenkel F., Moore S.S. & Stothard P. (2016) Genome-wide association for milk production and female fertility traits in Canadian dairy Holstein cattle. BMC Genetics 17, 75.

91. O’Halloran F., Berry D., Bahar B., Howard D., Sweeney T. & Giblin L. (2010) Polymorphisms in the bovine lactoferrin promoter are associated with reproductive performance and somatic cell count. Journal of Dairy Science 93, 1253-9.

92. Oikonomou G., Michailidis G., Kougioumtzis A., Avdi M. & Banos G. (2011) Effect of polymorphisms at the gene loci on reproduction, milk yield and lameness of Holstein cows. Research in Veterinary Science 91, 235-9.

93. Olsen H., Hayes B., Kent M., Nome T., Svendsen M., Larsgard A. & Lien S. (2011) Genome‐wide association mapping in Norwegian Red cattle identifies quantitative trait loci for fertility and milk production on BTA12. Animal Genetics 42, 466-74.

94. Olsen H., Hayes B., Kent M., Nome T., Svendsen M. & Lien S. (2010) A genome wide association study for QTL affecting direct and maternal effects of stillbirth and dystocia in cattle. Animal Genetics 41, 273-80.

95. Olsen H., Meuwissen T., Nilsen H., Svendsen M. & Lien S. (2008) Fine mapping of quantitative trait Loci on bovine chromosome 6 affecting calving difficulty. Journal of Dairy Science 91, 4312-22.

96. Ortega M.S., Denicol A.C., Cole J.B., Null D.J. & Hansen P.J. (2016) Use of single nucleotide polymorphisms in candidate genes associated with daughter pregnancy rate for prediction of genetic merit for reproduction in Holstein cows. Animal Genetics 47, 288-97.

97. Ortega M.S., Denicol A.C., Cole J.B., Null D.J., Taylor J.F., Schnabel R.D. & Hansen P.J. (2017) Association of single nucleotide polymorphisms in candidate genes previously related to genetic variation in fertility with phenotypic measurements of reproductive function in Holstein cows. Journal of Dairy Science 100, 3725-34.

98. Pausch H., Flisikowski K., Jung S., Emmerling R., Edel C., Götz K.-U. & Fries R. (2011) Genome-wide association study identifies two major loci affecting calving ease and growth-related traits in cattle. Genetics 187, 289-97.

99. Peters S.O., Kizilkaya K., ., Garrick D.J., Fernando R.L., Reecy J.M., Weaber R.L., Silver G.A. & Thomas M.G. (2013) Heritability and Bayesian genome-wide association study of first service conception and pregnancy in Brangus heifers. Journal of Animal Science 91, 605-12.

100. Pimentel E., Bauersachs S., Tietze M., Simianer H., Tetens J., Thaller G., Reinhardt F., Wolf E. & König S. (2011) Exploration of relationships between production and fertility traits in dairy cattle via association studies of SNPs within candidate genes derived by expression profiling. Animal Genetics 42, 251-62.

101. Purfield D.C., Bradley D.G., Evans R.D., Kearney F.J. & Berry D.P. (2015) Genome-wide association study for calving performance using high-density genotypes in dairy and beef cattle. Genetics Selection Evolution 47, 47.

102. Raven L.A., Cocks B.G., Kemper K.E., Chamberlain A.J., Vander Jagt C.J., Goddard M.E. & Hayes B.J. (2016) Targeted imputation of sequence variants and gene expression profiling identifies twelve candidate genes associated with lactation volume, composition and calving interval in dairy cattle. Mammalian Genome 27, 81-97.

103. Rychtářová J., Sztankóová Z., Kyselová J., Zink V., Štípková M., Vacek M. & Štolc L. (2014) Effect of DGAT1, BTN1a1, OLR1, and STAT1 genes on milk production and reproduction traits in the Czech Fleckvieh breed. Czech Journal of Animal Science 59, 45-53.

104. Sahana G., Höglund J.K., Guldbrandtsen B. & Lund M.S. (2015) Loci associated with adult stature also affect calf birth survival in cattle. Bmc Genetics 16, 47.

105. Sahana G., Nielsen U.S., Aamand G.P., Lund M.S. & Guldbrandtsen B. (2013) Novel harmful recessive haplotypes identified for fertility traits in Nordic Holstein cattle. Plos One 8, e82909.

106. Santos-Biase W.K.F., Biase F.H., Buratini Jr J., Balieiro J., Watanabe Y.F., Accorsi M.F., Ferreira C.R., Stranieri P., Caetano A.R. & Meirelles F.V. (2012) Single nucleotide polymorphisms in the bovine genome are associated with the number of oocytes collected during ovum pick up. Animal Reproduction Science 134, 141-9.

107. Saowaphak P., Duangjinda M., Plaengkaeo S., Suwannasing R. & Boonkum W. (2017) Genetic correlation and genome-wide association study (GWAS) of the length of productive life, days open, and 305-days milk yield in crossbred Holstein dairy cattle. Genetics & Molecular Research 16.

108. Sasaki S., Ibi T., Ikeda S. & Sugimoto Y. (2014) A genome‐wide association study reveals a quantitative trait locus for age at first calving in delta/notch‐like EGF repeat containing on chromosome 2 in J apanese B lack cattle. Animal Genetics 45, 285-7.

109. Sasaki S., Ibi T., Kojima T. & Sugimoto Y. (2016) A genome‐wide association study reveals a quantitative trait locus for days open on chromosome 2 in J apanese B lack cattle. Animal Genetics 47, 102-5.

110. Sassi N.B., González-Recio Ó., De Paz-Del Río R., Rodríguez-Ramilo S.T. & Fernández A.I. (2016) Associated effects of copy number variants on economically important traits in Spanish Holstein dairy cattle. Journal of Dairy Science 99, 6371-80.

111. Schnabel R.D., Sonstegard T.S., Taylor J.F. & Ashwell M.S. (2005) Whole-genome scan to detect QTL for milk production, conformation, fertility and functional traits in two US Holstein families. Animal Genetics 36, 408-16.

112. Schulman N.F., Sahana G., Iso-Touru T., Mckay S.D., Schnabel R.D., Lund M.S., Taylor J.F., Virta J. & Vilkki J.H. (2011) Mapping of fertility traits in Finnish Ayrshire by genome-wide association analysis. Animal Genetics 42, 263-9.

113. Schulman N.F., Sahana G., Lund M.S., Viitala S.M. & Vilkki J.H. (2008) Quantitative trait loci for fertility traits in Finnish Ayrshire cattle. Genetics Selection Evolution 40, 195.

114. Seidenspinner T., Tetens J., Habier D., Bennewitz J. & Thaller G. (2011) The placental growth factor (PGF)–a positional and functional candidate gene influencing calving ease and stillbirth in German dairy cattle. Animal Genetics 42, 22-7.

115. Shirasuna K., Kawashima C., Murayama C., Aoki Y., Masuda Y., Kida K., Matsui M., Shimizu T. & Miyamoto A. (2010) Relationships between the first ovulation postpartum and polymorphism in genes relating to function of immunity, metabolism and reproduction in high-producing dairy cows. Journal of Reproduction and Development, 1010290314-.

116. Sikora K.M., Magee D.A., Berkowicz E.W., Berry D.P., Howard D.J., Mullen M.P., Evans R.D., MacHugh D.E. & Spillane C. (2011) DNA sequence polymorphisms within the bovine guanine nucleotide-binding protein Gs subunit alpha (Gsα)-encoding (GNAS) genomic imprinting domain are associated with performance traits. Bmc Genetics 12, 4.

117. Silveira P.A.S., Butler W.R., Lacount S.E., Overton T.R., Barros C.C. & Schneider A. (2019) Polymorphisms in the anti-oxidant paraoxonase-1 (PON1) gene associated with fertility of postpartum dairy cows. Theriogenology 125, 302-9.

118. Tang K.Q., Li S.J., Yang W.C., Yu J.N., Han L., Li X. & Yang L.G. (2011) An MspI polymorphism in the inhibin alpha gene and its associations with superovulation traits in Chinese Holstein cows. Molecular Biology Reports 38, 17-21.

119. Tang K.Q., Yang W.C., Li S.J. & L-G Y. (2013) Polymorphisms of the bovine growth differentiation factor 9 gene associated with superovulation performance in Chinese Holstein cows. Genetics & Molecular Research Gmr 12, 390-9.

120. Tiezzi F., Arceo M.E., Cole J.B. & Maltecca C. (2018) Including gene networks to predict calving difficulty in Holstein, Brown Swiss and Jersey cattle. BMC genetics 19, 20.

121. Trakovická A., Moravčíková N. & Kasarda R. (2013) Genetic polymorphisms of leptin and leptin receptor genes in relation with production and reproduction traits in cattle. Acta Biochimica Polonica 60, 783-7.

122. Vaiciunas A., Coutinho L.L., Meirelles F.V., Pires A.V. & Silva L.F.P. (2008) Leptin and hypothalamic gene expression in early-and late-maturing Bos indicus Nellore heifers. Genetics and Molecular Biology 31, 657-64.

123. Wang X., Maltecca C., Tal-Stein R., Lipkin E. & Khatib H. (2008) Association of bovine fibroblast growth factor 2 (FGF2) gene with milk fat and productive life: An example of the ability of the candidate pathway strategy to identify quantitative trait genes. Journal of Dairy Science 91, 2475-80.

124. Waters S., McCabe M., Howard D., Giblin L., Magee D., MacHugh D. & Berry D. (2011) Associations between newly discovered polymorphisms in the Bos taurusgrowth hormone receptor gene and performance traits in Holstein–Friesian dairy cattle. Animal Genetics 42, 39-49.

125. Wei Y., Liu Guifen, Haijian C., Xiaomu L., Yuni Y. & Enliang S. (2015) Genetic Variation in GDF9、BMP15、FSHβ、FSHR Gene and Its Relationship with Twinning Trait in Cattle. Grass-feeding Livestock, 33-9.

126. Weller J.I., Golik M., Seroussi E., Ron M. & Ezra E. (2008) Detection of quantitative trait loci affecting twinning rate in Israeli Holsteins by the daughter design. Journal of Dairy Science 91, 2469-74.

127. Wickramasinghe S., Rincon G. & Medrano J. (2011) Variants in the pregnancy-associated plasma protein-A2 gene on Bos taurus autosome 16 are associated with daughter calving ease and productive life in Holstein cattle. Journal of Dairy Science 94, 1552-8.

128. Xing J., Ge X., Zhao J. & Liu Y. (2003) General situation and prospect of research on early pregnancy diagnosis technology for dairy cows. LIVESTOCK AND POULTRY INDUSTRY, 18-9.

129. Yang C., Zheng H., Shang J. & Huang F. (2016) Genetic polymorphism of FSHR, INHA, LHCGR and OPN Loci and their association with supemvulation traits in buffalo. Animal Husbandry & Veterinary Medicine 48, 75-8.

130. Yang W.C., Li S.J., Chen L. & Yang L.G. (2014) Polymorphism of the inhibin βA gene and its relationship with superovulation traits in Chinese Holstein cows. Genetics and Molecular Research 13, 269-75.

131. Yang W.C., Li S.J., Tang K.Q., Hua G.H., Zhang C.Y., Yu J.N., Li H. & Yang L.G. (2010) Polymorphisms in the 5′ upstream region of the FSH receptor gene, and their association with superovulation traits in Chinese Holstein cows. Animal Reproduction Science 119, 172-7.

132. Yang W.C., Tang K.Q., Li S.J. & Yang L.G. (2011) Association analysis between variants in bovine progesterone receptor gene and superovulation traits in Chinese Holstein cows. Reproduction in Domestic Animals 46, 1029-34.

133. Yang W.C., Yang L.G., Riaz H., Tang K.Q., Chen L. & Li S.J. (2013) Effects in cattle of genetic variation within the IGF1R gene on the superovulation performance and pregnancy rates after embryo transfer. Animal Reproduction Science 143, 24-9.

134. Yu Y., Pang Y., Zhao H., Xu X., Wu Z., An L. & Tian J. (2012) Association of a missense mutation in the luteinizing hormone/choriogonadotropin receptor gene (LHCGR) with superovulation traits in Chinese Holstein heifers. Journal of animal science and biotechnology 3, 35.

135. Zhang Q., Guldbrandtsen B., Thomasen J.R., Lund M.S. & Sahana G. (2016) Genome-wide association study for longevity with whole-genome sequencing in 3 cattle breeds. Journal of Dairy Science 99, 7289-98.
